# Cryo-electron Microscopy of Adeno-associated Virus

**DOI:** 10.1021/acs.chemrev.1c00936

**Published:** 2022-05-16

**Authors:** Scott M. Stagg, Craig Yoshioka, Omar Davulcu, Michael S. Chapman

**Affiliations:** † Department of Biological Sciences, Florida State University, Tallahassee, Florida 32306, United States; ‡ Institute of Molecular Biophysics, Florida State University, Tallahassee, Florida 32306, United States; § Department of Biomedical Engineering, Oregon Health & Science University, Portland Oregon 97239, United States; ∥ Environmental Molecular Sciences Laboratory, Pacific Northwest National Laboratory, 3335 Innovation Boulevard, Richland, Washington 99354, United States; ⊥ Department of Biochemistry, University of Missouri, Columbia, Missouri 65211, United States

## Abstract

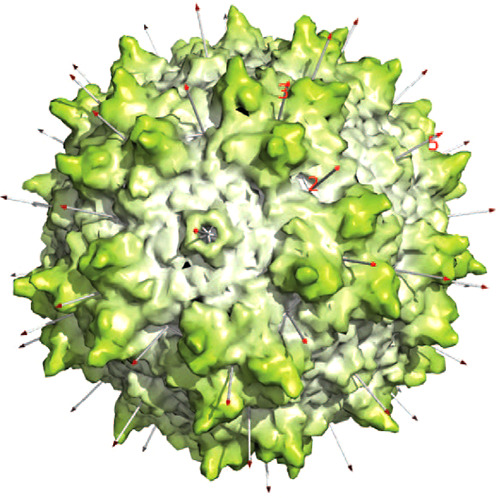

Adeno-associated virus (AAV) has a single-stranded DNA genome encapsidated in a small icosahedrally symmetric protein shell with 60 subunits. AAV is the leading delivery vector in emerging gene therapy treatments for inherited disorders, so its structure and molecular interactions with human hosts are of intense interest. A wide array of electron microscopic approaches have been used to visualize the virus and its complexes, depending on the scientific question, technology available, and amenability of the sample. Approaches range from subvolume tomographic analyses of complexes with large and flexible host proteins to detailed analysis of atomic interactions within the virus and with small ligands at resolutions as high as 1.6 Å. Analyses have led to the reclassification of glycan receptors as attachment factors, to structures with a new-found receptor protein, to identification of the epitopes of antibodies, and a new understanding of possible neutralization mechanisms. AAV is now well-enough characterized that it has also become a model system for EM methods development. Heralding a new era, cryo-EM is now also being deployed as an analytic tool in the process development and production quality control of high value pharmaceutical biologics, namely AAV vectors.

## 1 Introduction

**Figure 1:**
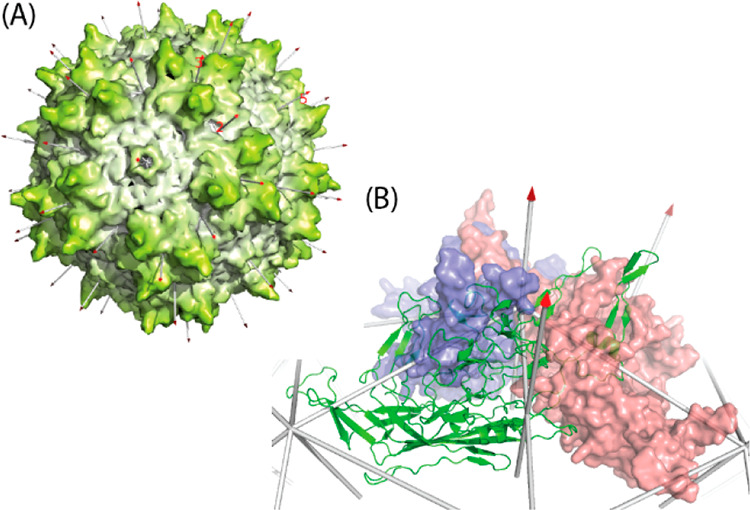
Capsid structure. (A) The surface of AAV2 is viewed approximately down a 5-fold axis.^1^ Data from both a mouse parvovirus and AAV suggests that an opening of the pore allows extrusion of the VP1-encoded phospholipase A2 (PLA_2_) domain for endosomal escape and for DNA entry/exit.^8−12^ Partially ordered density along the 5-fold pore in the AAV8 crystal structure suggests that some N-termini are external, as in several autonomous parvoviruses, connected by polypeptide chain running through the pore to the start of the β-barrel on the inner surface of the capsid.^13−16^ Above and to the right of the 5-fold, a 3-fold axis is surrounded by spikes that figure prominently in cellular entry and immune neutralization. (B) Three subunits intertwine around one of the 3-fold axes. The green ribbon shows the secondary structure common to VP1–3, dominated by the β-barrel on the inside surface of the capsid. In parvoviruses, the loops between β-strands are of unusual length, containing their own secondary structures and interacting with loops of neighboring subunits to form functionally important surface topologies that are distinctive to the major parvovirus genera.^1,14,17−19^ The gross surface features are more conserved between AAVs (than between parvoviral families), but structural differences are sufficient to account for distinctive virus–host interactions.

Adeno-associated virus (AAV) is a human small virus (25 nm diameter) from the parvovirus family with a single-stranded DNA genome, surrounded by a protein shell.^1^ This outer capsid is comprised of 60 viral protein (VP) subunits in an icosahedral assembly (Figure 1).^1^ Three variants of the capsid protein, VP1–3, are generated from the same transcript through alternate in-frame splicing/initiation sites.^2,3^ VP1, 2, and 3 are present in approximately 1:1:10 ratio,^4^ all sharing a β-barrel fold that is common among virus structures,^5,6^ but VP1–3 differ at their N-termini. The N-terminal additions in VP1 and 2 have been largely refractory to structure analysis, so little is known of their disposition. The core amino acids comprising residues ∼220–735 (conventionally numbered starting at the N-terminus of VP1), from the region common to all VP1–3 proteins, are mostly well-ordered, conform to the 60-fold *T* = 1 icosahedral symmetry and are the region seen in the structures determined to date.^1,7^

AAVs belong to the *Dependovirus* genus of the *Parvovirinae* subfamily that infect mammals.^20^ Dependoviruses differ from “autonomous” *Parvovirinae*, because their replication depends upon coinfection with a “helper” virus.^21^ Indeed, AAV is so named because it was first characterized as a contaminant in early studies of adenovirus, although other viruses, such as herpes, can also provide needed replication machinery.^21−24^ So, AAV’s name comes not from any similarity to adenovirus but from its parasitic dependence upon “helper” functions provided by adeno- or other viruses.^25^ Dependent viruses, like AAV, are sometimes termed “defective” or “satellite”. In its natural life cycle, AAV’s initial infection is latent with a mixture of site-specifically or randomly integrated DNA or episomal retention (particularly for vectors).^4,26−31^ Virus replication is then rescued upon coinfection by the helper virus. AAVs are mostly regarded as nonpathogenic, unlike their disease-causing parvovirus cousins, largely because the pathogenic effects of AAV are not distinguishable from those of the helper virus.^32,33^ The lack of disease was touted during early development of AAV as a transducing vector for gene therapy.^34,35^ This has become controversial first with animal studies and then clinical studies showing 7–21% of hepatocellular carcinoma (HCC) patients had AAV sequences inserted into known proto-oncogenes, leading to elevated expression.^36−39^ There has been vigorous debate about the significance of these results, regarding etiology of natural infection, whether a causal link had been established, and whether injected vectors could have similar effects.^40−42^ The prevalent view is that heightened HCC in those with chronic liver disease may warrant screening before AAV-mediated gene therapy, but otherwise a low risk of a serious adverse event is usually offset by the benefit of the therapy.^43−45^

Interest in AAV stems mostly from its use as a delivery vector. Recombinant vectors (rAAV) differ from their wild-type forebears (wtAAV) in replacement of most of the viral DNA with that of a transgene expressing a needed protein (or editing a gene correction).^34,46−54^ (rAAV DNA sequences usually retain just the viral inverted terminal repeats, ITRs, 145 base sequences at each end of the transfer vector that are needed for self-primed synthesis of the DNA second strand and are recognized as the signal for DNA encapsidation within protein capsids.) Thus, in comparing wtAAV and rAAV, we might expect the interior nucleic acid to be quite different, but methods of producing rAAV have been designed to mimic a wt-like configuration of the protein capsid. The most widely used rAAV production methods use triple transfection of human cells in which (adenoviral-derived) helper functions, capsid genes, and the transgene are provided on different plasmids, avoiding adenoviral contamination.^55−57^ Recombinant baculovirus expression vectors (BEV) have been developed for production in Sf9 insect cells, primarily to eliminate human DNA from clinical preparations, and these have been optimized to better reflect the natural abundance of VP1–3.^58−61^

The greatest investments in AAV vectors have been with gene replacement treatments for monogenic genetic diseases, most famously resulting in the recently FDA-approved treatment for the debilitating and fatal disease, spinal muscular atrophy (SMA^62−64^), with other gene therapies in the clinical trial pipeline for a range of diseases including hemophilia.^65−67^ There is also much excitement for the potential of gene editing using AAV vectors.^52,66,68,69^ Another application has been in development for 15 years, vaccination against viral pathogens through delivery of a vector encoding a neutralizing antigen,^70−74^ which has seen resurgent interest with the successful testing of COVID-19 vaccines in nonhuman primates.^75^

Structural studies started with crystallization of wtAAV infectious particles produced by transfection of HeLa cells with a plasmid clone of the virus.^76−78^ The scale up from microgram to milligram quantities of wtAAV had been a rate-limiting challenge in structure determination.^78^ Alternative sample preparations have now been developed that are more expedient. Despite differing nucleic acid content, high-resolution structures of the symmetrical part of the capsid have been mostly the same.^79−83^ For structural studies, it is now more usual to use a simplified BEV system, omitting the transgene construct, to produce “empty” virus-like particles (VLP) at high yield.^84^ While these particles contain no wt viral or transgene DNA, they are often at least partially filled, one assumes serendipitously, with cellular nucleic acids. The wtAAV or BEV-produced VLP were needed when structural biology demanded milligram quantities. It is now exciting to see the success of high-resolution cryo-EM with the much smaller quantities derived from the triple transfection methods of preparing rAAV vectors, sometimes then purified only by Cs-gradient ultracentrifugation.^82^ There had been indications of capsid plasticity, dependent on DNA content, from subnanometer cryo-EMs of AAV1 and AAV2.^85^ However, classification of DNA-containing and empty particles from high resolution images of triple transfection vectors for four rAAV serotypes revealed no significant differences.^86^ Thus, except as noted, capsids produced in the different ways will be regarded as equivalent.

**Figure 2:**
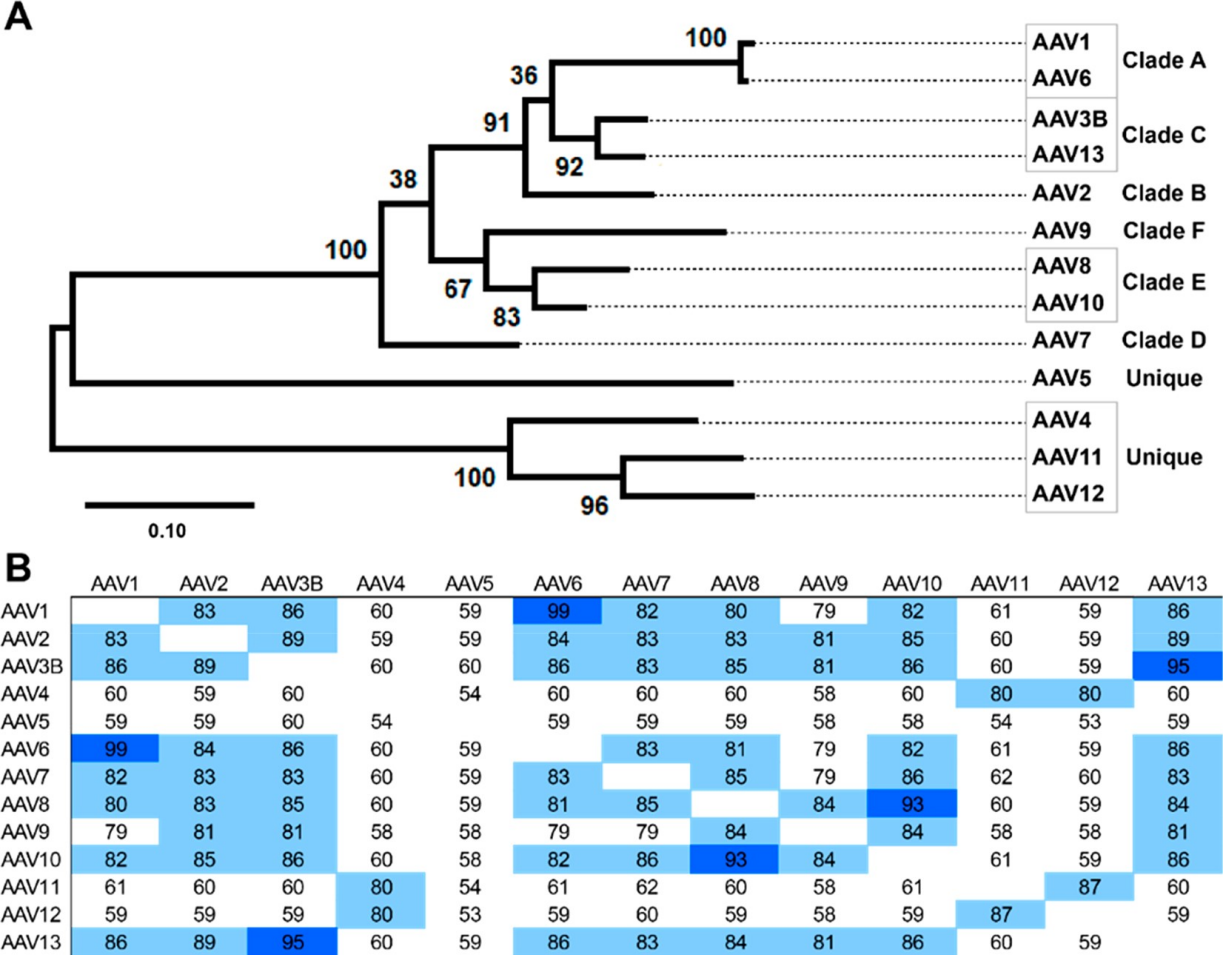
Phylogenetic relationships between primate AAV VP3 major capsid proteins. (A) A maximum likelihood tree, showing, at each node, the bootstrap probability based on 500 replicates. The scale bar shows the fraction of amino acid substitutions per branch length. Representative serotypes are shown, grouped by clade, where applicable. VP3 sequences were curated manually from AAV1–13 VP1 (AAV1–13, GenInfo identifiers, respectively: NP_049542.1, YP_680426.1, AAB95452.1, NP_044927.1, YP_068409.1, AAB95450.1, YP_077178.1, AAS99264.1, AAT46337.1, AAT46339.1, ABI16639.1, and ABZ10812.1) and aligned with MUSCLE.^103^ The tree was generated using MEGA X.^104^ (B) Amino acid identities (%) based on pairwise alignment. Dark-blue highlights >90% identity; light-blue highlights 80–90% identity.

Beyond the basic structure of the icosahedral assembly, cryo-EM has mostly been applied to understanding the early steps of infection and immune recognition. Over 130 AAV variants have been identified with human or other primate hosts.^87,88^ These can be grouped into eight major named and unnamed clades (Figure 2) with, as detailed later, representative structures for each. Each group contains one or more serotypes that are antigenically distinct, *i.e.*, immunity elicited to one virus does not confer immunity to other serotypes. The immune responses to gene therapy vectors (and transduced cells) are critical determinants of treatment efficacy, while immune toxicity is the major consideration in safety profiles.^89−92^ Thus, wide-ranging studies into diverse immune mechanisms continue.^91,93^ Structure and AAV immunology have intersected primarily with the adaptive humoral (antibody) response. While one might expect that surface properties would have been driven evolutionarily by selection of attachment and receptor interactions favorable for host range, greater diversity has likely been driven, as in other parvoviruses, by selective pressure to evade the recognition of known immunogens.^94−102^ Comparative structural studies (below) have been undertaken for insights into the functional phenotypes of different serotypes, while analyses of virus–antibody complexes provide fundamental insights into neutralization mechanisms and inspire the design of immune-evading rAAV vectors.

Cryo-EM has been central to re-evaluating AAV’s receptor-mediated cell entry. In 1998, heparan sulfate proteoglycan (HSPG) was identified as the receptor for AAV2.^105^ HSPG was also identified for AAV3 and 6, while other glycosaminoglycans were identified as “primary” receptors for other serotypes: sialic acid (SIA) terminated glycans for AAV4, 5, 1 and 6 and terminal galactose for AAV9.^106−110^ A number of membrane proteins, primarily tyrosine kinase receptors and integrins, were identified as coreceptors for different serotypes, helping to mediate endosomal entry: fibroblast growth factor receptor (FGFR), hepatocyte growth factor receptor (HGFR aka c-Met), platelet-derived growth factor receptor (PDGFR), laminin, and integrins α_V_β_1_ and α_V_β_5_.^111−118^ Recently, genome-wide screens were used to identify host cell factors most essential for viral transduction, and none of these coreceptors were implicated by these screens.^119−121^ There has been no evidence of direct physical interactions between the previously reported coreceptors and AAV, and in several cases, targeted CRISPR knockout had little impact.^119^ The coreceptors were identified in an era when viral receptor identification was challenging and sometimes controversial. It can be difficult to determine the roles of host molecules if there are redundant entry pathways or host proteins that affect viral transduction indirectly by modulating the cell state. Nevertheless, roles ascribed to previously identified coreceptors should be revisited given the technical advances such as isogenic CRISPR-Cas9 knockouts that can provide more definitive insights into function. The genome-wide screens have shown as necessary several proteins involved in synthesis of extracellular glycans and in endosomal trafficking, but repeatedly a previously uncharacterized membrane protein, now called adeno-associated virus receptor (AAVR), has been among the top hits, and its role in cell entry and trafficking for most AAV serotypes has been confirmed in multiple ways.^119,120^ Other membrane proteins have also been implicated repeatedly, such as GPR108, TM9SF2, and ATP2C1.^119−122^ Some might prove to have direct interactions, but characterization is only just beginning, with GPR108 thought to have a role downstream of AAVR, perhaps as AAV escapes from endosomes.^121^ In summary, of host molecules implicated, AAVR has greatest impact upon transduction and is considered one (of perhaps several) membrane proteins key in cell entry and trafficking. As further detailed below, glycan interactions are less specific than once thought, and they should be considered to be attachment factors (rather than entry receptors) tethering AAV to the cell surface to enhance the likelihood of AAV binding to AAVR for productive endocytosis.

### 1.1 Cryo-EM Replacing Crystallography

**Figure 3:**
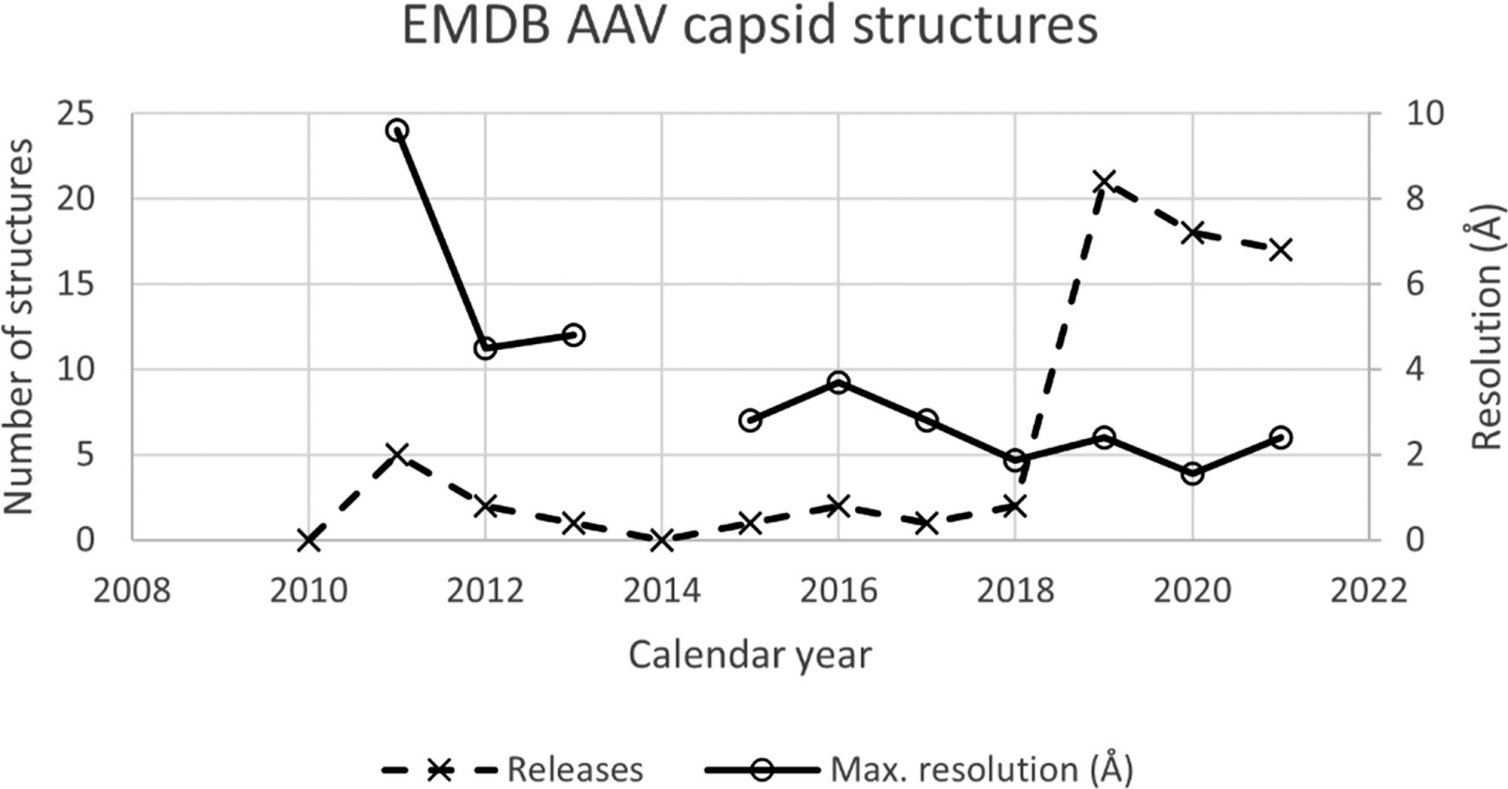
Progress in AAV cryo-EM. Through 2014, cryo-EM complemented higher resolution X-ray diffraction with studies of complexes, transitions, or less-ordered components that were not amenable to crystallography. 2015 brought the first structure beyond 3 Å, a resolution that supports the building of atomic models.^123^ Over a three-year period, the enabling EM technology was more broadly disseminated, leading to substantial growth in 2020. Also shown is the steady improvement in the highest resolution attained in each year. While it still requires special care to reach the highest 1.5–1.9 Å resolutions, it is becoming more routine to reach the 2–3 Å regime from which atomic models are readily derived.^128,129^

X-ray crystallography provided the first high-resolution AAV structure in 2002 and reigned supreme until the first cryo-EM structure at a comparable 2.8 Å resolution in 2015.^1,123^ So swift has been the takeover (Figure 3) that the last capsid PDB entry solved by X-ray diffraction was released in 2016.^124^ At the time of writing, 51 of 69 (74%) atomic models at the PDB are EM-derived.^125^ It is 61 of 79 (77%) when including reconstructions at lower (nanometer) resolutions, likely an underestimate because of the higher fraction of low-resolution structures that have not been deposited at the EMDB.^126^ The AAV capsid is the focus of this review, but the DNA replication of AAV is now also being studied by mid-resolution cryo-EM.^127^

## 2 AAV Structural Virology

This section reviews the advances in AAV virology coming through EM. Discussions of a technological nature are cross-referenced to the following section.

### 2.1 Capsid Structures of Representative Serotypes

**Figure 4:**
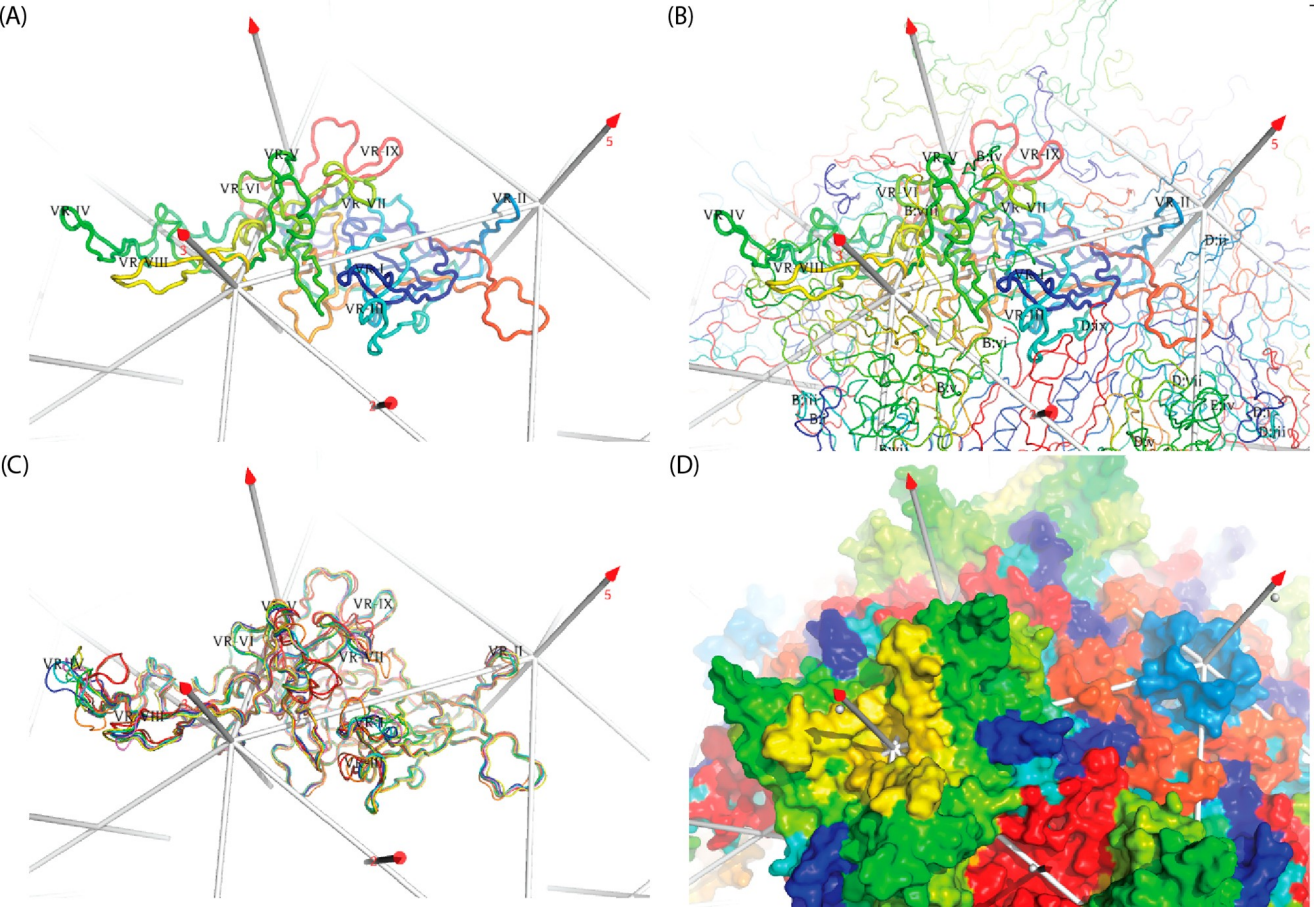
VP3 capsid protein subunit structure. The view is from the outside, looking down an icosahedral 2-fold, with a 3-fold left, and a 5-fold right. In traces A–C, the conserved β-barrel is behind the outer surface loops in the foreground. (A, B, D) Rainbow coloring is from blue (N-terminus) to red (C-terminus). (A) A subunit from the AAV2 crystal structure^1^ is annotated by sequence-variable region (VR).^148^ VR-IV through VR-VIII are all contributed by a long loop between β-strands G and H. (B) Neighboring subunits are added (thinner trace), with VRs of close neighbors intertwined and annotated in abbreviated form (chain-id:loop no.). (C) Structures of representative serotypes 2–9 and 11 are overlaid, colored by order in the AAV phylogenetic tree (Figure 2): AAV6 in violet, AAV3 in blue, AAV2 in cyan, AAV9 in green, AAV8 in lime, AAV7 in yellow, AAV5 in orange, AAV4 in brown, and AAV11 in red. AAV6, AAV3B, AAV2, AAV9, AAV8, and AAV4 are the crystal structures (PDB 3shm,^81^3kic,^149^3ux1,^150^2qa0,^13^ and 2g8g(148)), while AAV7, AAV5, and AAV11 are EM structures that are new (7jot, 7l6f(86)) or at appreciably higher resolution (7kp3^147^). From this, we see that, in approximate order, VR-IV, VR-V, VR-VII, VR-I, and then VR-III exhibit the most structural diversity, with VR-IX, VR-VI, and VR-II more conserved. The outliers are usually AAV5, AAV4, and AAV11 but differing by location. The tip of VR-IV extends out in most serotypes, but turns tangentially in one direction for AAV4 and AAV11 and in the opposite direction for AAV5. At the C-terminal end of VR-V, just AAV4 and 11 have a six-residue insertion, whereas much of the AAV5 loop is displaced ∼2 Å in the opposite direction. At VR-VII, a single insertion in AAV4/11 moves the base of the loop toward the spike (relative to most serotypes), but, in AAV5, a three-residue insertion extends the loop 6 Å further from the spike. VR-I loops differ in length by five residues with AAV4/11 the shortest, a single insertion point for clades A–F with inserts of diverse conformation and an insertion point for AAV5 two residues later. Finally, VR-III is well conserved for clades A–F, but has two-residue insertions for AAV5 and for AAV4/11, the latter displaced 3 Å further. (D) With AAV2 as an example, the surface is rainbow-colored as in (A), showing VR-I as blue, VR-II as cyan, VR-III as aquamarine, VR-IV as dark-green, VR-V as light-green, VR-VI as lime, VR-VII as chartreuse, VR-VIII as yellow, the HI loop as brown, and VR-IX as red.

As evident from Table 1, there is rapid growth in available structures, particularly in the 2.5–3.0 Å resolution ranges that are sufficient to trace backbone and model side chains but have become accessible to standard cryo-EM approaches. We now have broader and finer-grained representation, in structure, of the phylogenetic tree (Figure 2) through purposeful discovery directed at missing gaps.^86,135,141^ Representative structures are overlaid in Figure 4, which also illustrates the interdigitation of loops from adjacent subunits in the viral assembly. We now also have structures that have leap-frogged, in resolution, prior crystal structures of native state AAVs, often as unheralded biproducts, as the “controls” in studies of ligand- or environmentally induced conformational change, *e.g.*, AAV5 and AAV9.^144,147^ Without ignoring the substantial efforts previously expended in “foundation” structures, checks of the current database contents frequently show depositions of improved resolution.

**Table 1:** Notable AAV Cryo-EM Structures

serotype	date	PDB	EMDB	resolution (Å)	type	notes, citation
*AAV2*	2001	n/a	1907	10.5	VLP	(130)
*AAV2*	2005	n/a		10.1	VLP	(131)
*AAV2*	2005	n/a		10.3	VLP	heat-treated^131^
*AAV2 mutant*	2005	n/a		10.4	VLP	VP1-deleted^131^
*AAV2 mutant*	2005	n/a		10.1	VLP	VP2-deleted^131^
*AAV1*	2011	n/a	1836–1839	9.6	vectors	varying DNA^85^
*AAV-DJ*^a^^b^	2012	3J1Q	5415	4.5	chimeric VLP	(132)
*AAV2*	2016	5IPI	8099	3.8	VLP	(133)
*AAV2*	2016	5IPK	8100	3.7	R432A VLP	(133)
*AAV3B*	2019	n/a	20625	3.42	vector	with DNA^134^
*AAV3B*	2019	n/a	20624	3.26	vector	empty^134^
*AAV8*	2020	6V1Z	21020	3.77	vector	full, clade E^135^
*AAV8*	2020	6V1T	21017	3.08	vector	empty, clade E^135^
*AAVrh.10*	2020	6V10	21004	2.98	vector	full, clade E^135^
*AAVrh.10*	2020	6V12	21010	2.75	vector	empty, clade E^135^
*AAVrh.39*	2020	6V1G	21011	3.58	vector	full, clade E^135^
*AAVrh.39*	2020	6O9R	0663	3.39	vector	empty, clade E^135^
*AAV8*	2020	6PWA	20502	3.3	vector	full from HEK293^136^
*AAV8*	2020	6U2V	20626	3.6	BEV	full from sf9^136^
*AAV8*	2020	6U20	20615	3.3	vector	empty, HEK293^136^
*AAV8*	2020	6UBM	20710	3.3	BEV	empty from sf9^136^
*AAV1*	2019	6JCR	9795	3.07	vector	(137)
*AAV2.5*^b^	2018	6CBE	7452	2.78	vector	(137,138)
*AAV9_L001*^b^	2019	6NXE	0535	3.12	provector	(138,139)
*AAV2.7m8*^b^	2020	6U0R	20609	2.9	VLP	(139,140)
*AAVhu.37*	2020	6U95	20693	2.56	vector	clade E^140,141^
*AAVhu69/AAVv66*	2020	6U3Q	20630	2.46	vector	(142)
*BtAAV-10HB*	2020	6WFT	21656	3.03	vector	bat, with DNA^142,143^
*BtAAV-10HB*	2020	6WFU	21657	3.03	vector	bat, empty^143^
*AAV7*	2020	7JOT	22412	2.7		n/a
*AAV7*	2021	7L5Q	23190	2.96	vector	empty^86^
*AAV7*	2021	7L5U	23189	3.16	vector	with DNA^86^
*AAV11*	2021	7L6E	23202	3.15	vector	with DNA^86^
*AAV11*	2021	7L6F	23203	2.86	vector	empty^86^
*AAV12*	2021	7L6A	23200	2.67	vector	with DNA^86^
*AAV12*	2021	7L6B	23201	2.54	vector	empty^86^
*AAV13*	2021	7L6H	23204	3.0	vector	with DNA^86^
*AAV13*	2021	7L6B	23205	2.76	vector	empty^86^
*AAV2*	2020	6U0Y	20610	3.03	VLP	n/a
*AAV9*^a^	2021	7MT0	23973	2.82	VLP	pH 7.4^144^
*AAV9*^a^	2021	7MTG	23986	2.67	VLP	pH 6.0^144^
*AAV9*^a^	2021	7MTP	23993	2.79	VLP	pH 5.5^144^
*AAV9*^a^	2021	7MTW	23999	2.99	VLP	pH 4.0^144^
*AAV9-PHP.B*^b^	2021	7RK8	24494	2.27	vector	(145)
*AAV1-PHP.B*^b^	2021	7RK9	24495	2.32	vector	(145)
*AAVhum.8*^b^	2021	7LTM	23516	2.49	vector	(146)
*AAV2 mutant*	2018	6E9D	9012	1.86	L336C	image data: EMPIAR-10202 ^129^
*AAV5*	2020	7KP3	22987	2.1	VLP	(147)
*AAV-DJ*^b^	2020	7KFR	22854	1.56	chimeric VLP	image data: EMPIAR-10551^128^

a Complexes are tabulated separately, sometimes at higher resolution;

b non-natural engineered vectors.

**Figure 5:**
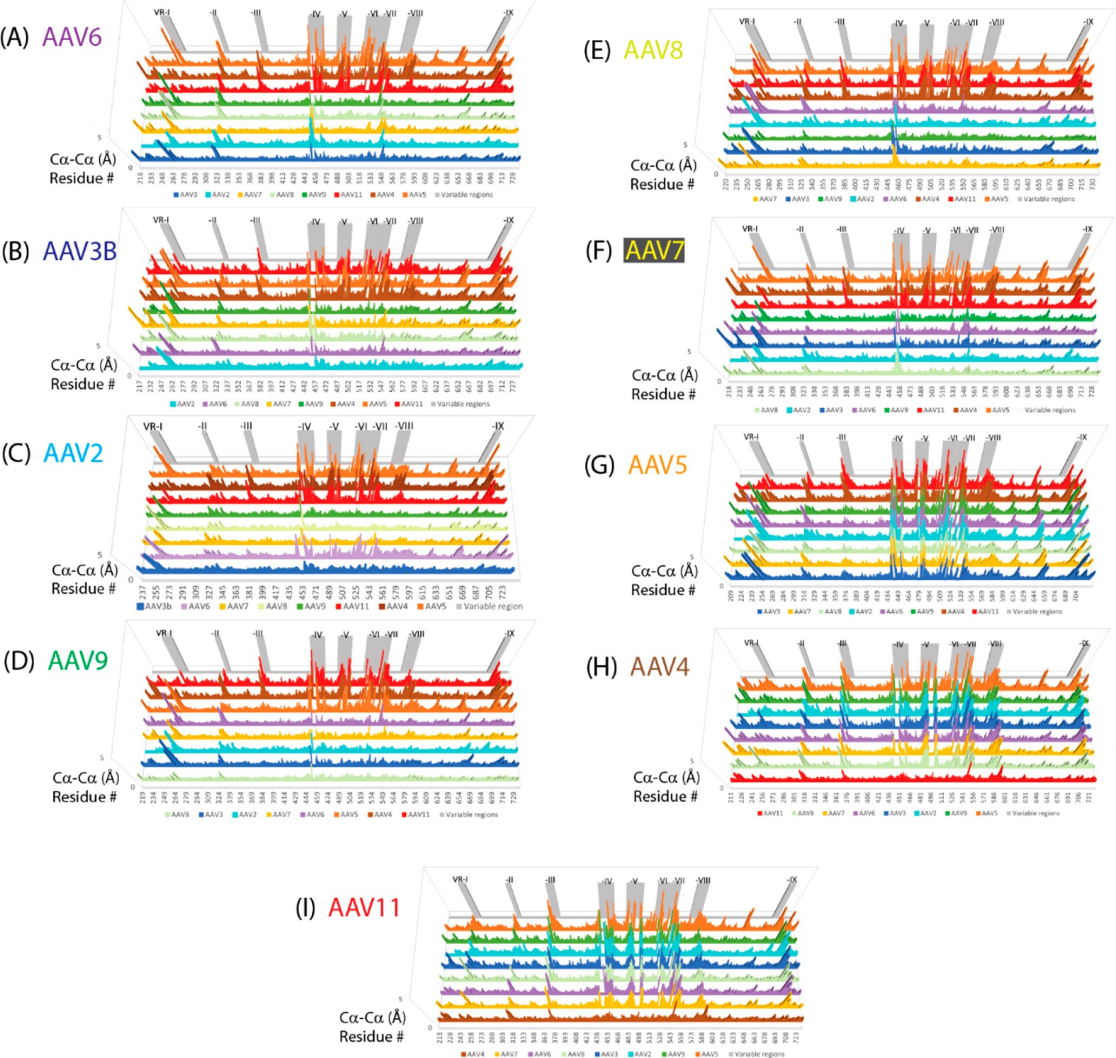
Structural variation among representative serotypes. Each panel shows the C_α_–C_α_ distance by residue number for the serotype labeled versus representatives of each phylogenetic group. The panels are ordered according to the phylogenetic tree in Figure 2 (so similar serotypes are clustered together) and with color coding of serotypes as in Figure 4C. In each panel, the serotype histograms are ordered by decreasing sequence identity from bottom to top. The nine variable regions (VR-I through VR-IX) are shaded gray in the backdrop of each panel. It is clear that the structural differences are greatest in the VRs. Between pairs of related serotypes, some of the VRs have diverged while others remain quite similar, generally with the number of diversified VRs increasing with evolutionary distance.

**Figure 6:**
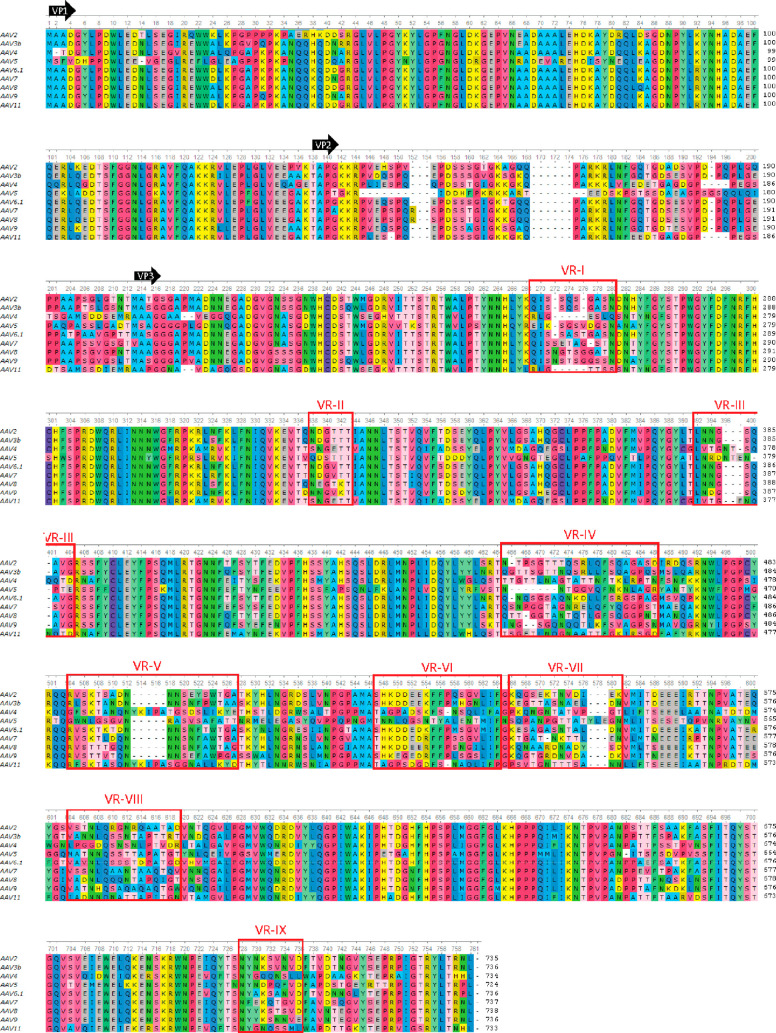
Representative AAV sequences aligned. The N-terminal amino acids of VP1, VP2, and VP3 are marked with black arrows. Variable regions (VRs^148^) are boxed and amino acids colored by type.

The recent study of Mietzsch *et al.* added the structures of four serotypes, all prepared as transducing vectors and yielding separate DNA-containing and empty-particle reconstructions following 2D classification.^86^ As in previous structures, maps are interpretable for the common part of VP1/2/3, actually starting at about residue 15 of VP3. Usually, the backbone is traceable through to the C-terminus, but in AAV7, as in AAVrh.10 and AAVrh.39, a short GGTxG sequence at the tip of a loop (VR-IV) is flexible and disordered.^86,135^ Differences between full and empty capsids are insignificant (<0.3 Å rmsd). Within the conserved β-barrel fold, rms differences with other serotypes (<0.5 Å) are within experimental error, and the new structures have overall surface topology that is similar to those solved before. Interest is really in the loops of less conserved sequence that decorate the surface (VR-I through VR-IX) (Figure 4, Figure 6).^148^ They differ by as much as 15 Å (C_α_) at VR-IV, with large (5–10 Å) differences also in VR-I, III, V, VI, VII, and IX, mostly between AAV11/12 and other serotypes (Figure 5) but also for AAV7 at VR-I and VR-IV.^86^ These are large changes in the loops that interact with receptors and antibodies.

### 2.2 Partially Ordered Elements

The comparison of capsid structures, above, focused on the symmetrical and ordered parts that typically start about 20 residues after the N-terminus of VP3. Not resolved by high resolution cryo-EM (or crystallography) are the less-ordered VP3 N-termini, and the ∼200 and ∼65 residues of the N-terminal extensions in VP1 and VP2, respectively. It is more challenging to resolve these elements, not only because of disorder but because VP1 and VP2 each constitute only 10% of the capsid proteins. On averaging according to the icosahedral symmetry, these regions are therefore systematically weaker. AAV cryo-EM had actually started at low (1–2 nm) resolution before high-resolution crystal structures were available.^130,151^ With publication of the first crystal structures, complementary EM studies focused on the parts that had not been resolved at high resolution: the interior DNA and the N-terminal extensions in VP1 and VP2.

**Figure 7:**
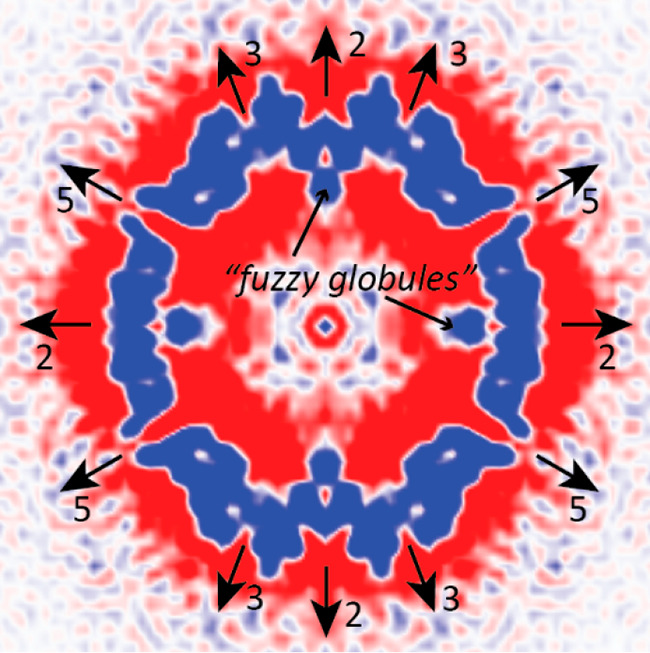
Cryo-EM reconstruction of AAV2 empty particles at 1 nm resolution.^130^ An equatorial section from EMD1907 has been rendered using PyMol with map values >1σ colored blue.^160^ Symmetry axes are indicated, as are “fuzzy globules”, features at the inner surface on 2-fold axes that were proposed to be the unique parts of VP1 or VP2 that do not correspond to atomic model in high-resolution structures.^130,131^

The VP1-unique region (VP1u) contains a phospholipase (PLA_2_) domain that is reported to be required for AAV to escape perinuclear endosomes, after conformational changes that release the domain from the interior.^152−157^ The role of the 65 amino acids at the N-terminus of VP2 (also within the C-terminal region of VP1u), immediately before the start of VP3, remains in question. Motifs containing basic residues had been implicated as nuclear localization signals (NLS), but subsequent studies left this less clear.^158,159^ Before the AAV2 crystal structure, diffuse protrusions, termed “fuzzy globules”, had been highlighted as extending inward from the inner capsid surface at the 2-fold axis (Figure 7).^130^ They were identified as the N-terminal extensions of VP3 (that are only present in VP1 or VP2 subunits) by comparing a nanometer-resolution cryo-EM reconstruction of empty AAV2 capsids to VP3 from the canine parvovirus crystal structure.^17,130^ The fuzzy globules were weak, but this is consistent with the N-terminal extensions present for only 20% of capsid proteins. Although not directly observable at nanometer resolution, it was proposed that a βA strand, N-terminal to the canonical viral jellyroll barrel, had a different configuration from seen in several parvoviruses, so that instead of running from the 5-fold pore, it would connect directly the “fuzzy globule” to the start of βB near the 2-fold.^130^ Others later hypothesized an unseen (disordered) connection from the “fuzzy globule” to the first resolved residues of the AAV2 crystal structure, near the 5-fold axis.^85^

Additional experiments supported designation of the “fuzzy globules” as VP1 or VP2. The feature was missing in mutant AAV2, with the unique parts of VP1 or VP2 deleted and also after a conformational change triggered to release VP1u from the inside.^131^ (In some parvoviruses, the transition can be triggered, *in vitro*, by an endosomal-like pH change, but for AAV a temperature jump is needed.^161^) However, later studies have been more equivocal. AAV1 is mostly similar to AAV2, but studies of rAAV1 with varying DNA content revealed no “fuzzy globules”.^85^

Support for the VP1/2u “fuzzy globules” did come from an AAV2 R432A mutant.^133^ The mutation decreases DNA-packaging efficiency by 5 logs. Normally, single-stranded DNA genomes are packaged into nucleoli-preassembled capsids using the AAV Rep52, Rep68, and Rep78 proteins expressed from the *rep* gene.^11,162−164^ Little is known about the structure of capsid-Rep protein assemblies, but there is mutational data for both AAV and minute virus of mouse that implicates binding near a 5-fold portal.^11,12^ The R432A mutation is one of two that are buried far away, near a 3-fold subunit interface. Conformational differences were expected in the R432A mutant because of a 10 °C thermal destabilization, and changes were seen to propagate toward the 5-fold.^133^ However, great care is needed when comparing atomic models, because a large part of reported differences (rmsd of 0.9–1.3 Å) is attributable to errors and ambiguities in fitting atomic models at intermediate resolution (see section 3.4.1). The βA strand was missing in the R432A map. The map showed four residues extending away from the βA−βB turn toward the 2-fold, the region where the “fuzzy globules” had been seen earlier, but only at 5 Å resolution, an indication of disorder, and nothing seen corresponded directly with the fuzzy globules.^133^

Recent cryo-electron tomography (ET) of complexes with receptor AAVR, at nanometer resolution, showed the fuzzy globules for AAV2 but not AAV5.^165^ Indicative of disorder, cryo-EM of the AAV2 complex at higher 2.4 Å resolution shows nothing of these features but does indicate that the predominant capsid protein conformation skips βA, merging in from the fuzzy globule area at the βA-βB turn.^83^ However, a reconstruction with a different receptor construct showed βA as the dominant configuration.^82^ It is very difficult to rationalize how receptor-binding far away on the outer surface (and the R432A mutation, above) affects the N-terminal conformation on the inner surface in some circumstances but not others. It is more likely that there is a finely balanced equilibrium between states that can be tipped by factors that are not understood or controlled in these experiments. In summary, several low-resolution studies have revealed the inner surface location where otherwise-unseen N-terminal regions of the capsid protein are sometimes located. The current characterization is partial, with parts of the N-terminal region and further alternative conformations yet to be discovered, and little understanding of the factors affecting the apparent heterogeneity.

### 2.3 Variant and Engineered rAAV Vectors

Cryo-EM took hold in AAV structure determination after many of the canonical natural serotypes had been solved crystallographically, and as attention was moving toward rAAV vectors engineered for altered cell tropism. First of these structures was AAV-DJ, which had been determined in native state at 4.5 Å resolution and was surpassed by a 3 Å resolution structure as an oligosaccharide complex and then a 1.56 Å uncomplexed structure (see section 3.2). AAV-DJ had been created from a library of randomly annealed hybrids of eight serotypes, with selection of variants to which hepatoma cells were permissive and that were resistant to pooled human antisera (IVIg).^166^ Although 92% identical to AAV2, AAV-DJ shows increased specificity for mouse liver, with up to 20-fold higher transduction, even following passive IVIg immunization. With an AAV9-like sequence in VR-I, the region between Asn_262_ and Ser_268_ constitutes the greatest deviation (4 Å) from AAV2. This hybrid was perhaps selected from the library as a direct result of IVIg escape selection because the AAV2 residues are prominent within the epitope of strongly neutralizing mAb A20.^167^ Here, AAV-DJ clashes with a superimposed Fab′ A20, explaining the negative A20 immunoblot.^167^ These VR-I residues might also be impacting cell tropism, because they are also within the footprint of AAVR on AAV2 (see below).^82,83^ Thus, the early structure of AAV-DJ, at intermediate resolution, provided clues on receptor-binding as well as showing a structural proof of principle that AAVs could be successfully modified for immune evasion.

AAV2.5 is a rationally engineered chimeric mutant that substituted five AAV1-like residues into an AAV2 background.^168^ This vector, intended for Duchenne muscular dystrophy treatments, was designed to retain AAV2 HSPG-binding with the addition of an AAV1-like muscle-tropic phenotype. The cryo-EM showed very clearly that the single-site mutations led to local changes of nearly 3 Å, rendering surface loop variable regions VR-I and VR-IV^148^ in conformations that were close to those of AAV1.^169^ This study demonstrated the potential, at least sometimes, of cryo-EM in a rational design work-flow and, in this case, how predictably changes to AAV capsids could be engineered through site-directed mutagenesis.

AAV9_L001 is a provector with an insertion in VR-IV on the 3-fold spike that can be cleaved by a matrix metalloproteinease (MMP) (before which it is transductionally inactive).^139^ Overall the structure was very similar to the parental AAV9. Backbone was traceable for three residues at the N-terminal end of the insertion, and four residues at the C-terminal end but was not resolved for the intervening 24 residues.^139^ It is not unexpected that a non-native peptide insertion, lacking “normal” interactions, is disordered. This disappointment aside, cryo-EM is affirming the potential for local engineering without disruption of the surrounding capsid assembly.

AAV2.7m8 has a 10-amino acid insertion in the surface loop known as VR-VIII that had been selected, in directed evolution, for efficiency in transducing mouse retina following intravitreal injection.^170^ The insertion point is between the two arginines of AAV2’s heparin-binding domain (HBD), disrupting binding to the HSPG attachment factor and leading to greater retinal penetrance.^170^ The map is weak at the insertion point, allowing modeling of backbone, but not side chains. Nevertheless, HBD residues Arg_585_/Arg_588_ were clear and unchanged from wtAAV2. So, the insertion likely occludes glycan access, reducing HSPG affinity, allowing the observed greater penetrance of the retina (by less hindered AAV).^140,170^ The structure also showed changes within the epitope of neutralizing monoclonal antibody, C-37B (see below), providing a rationale for reduced immune neutralization.^140,170^

AAV9 has been a promising vector for central nervous system (CNS) targeting, with higher expression in animal models.^171−173^ A “PHP.B” variant with improved CNS transduction was created by directed evolution, using a library of heptapeptide insertions at the site corresponding to AAV2’s HBD.^174−176^ Later, it would be found that the improved targeting depended on the Ly6a receptor and was specific to the C57BL/6J inbred mice used for *in vivo* selection.^177,178^ While dampening enthusiasm for translation of AAV-PHP.B into human use, the experience has been proof of principle that targeting could be modulated through peptide selection at an accessible site.^174,179^ Cryo-EM was used in establishing that the functional presentation of such a peptide depended on its structural context.^145^ The hepatapeptide was only partially ordered in the 2.3 Å structures of the AAV9 and AAV1 insertions, but the background structures were very similar to their parental types, ruling down the possibility that insertion had been disruptive upon the assembly. By grafting into AAV1 not only the heptapeptide, but by flanking AAV9 VR-VIII residues, a phenotype was obtained with Ly6a-mediated brain targeting but not the transcytosis needed for crossing the blood–brain barrier.^145^

The latest structure of an engineered vector is that of AAVhum.8.^146^ This variant of AAV8 is the product of directed evolution toward neutralizing immune escape, focusing, in turn, on different VR loops.^180^ Pleiotropic effects on cell tropism came with the fittest variants at VR-IV and VR-VIII, VR-VIII with glycan-binding basic amino acids, and VR-IV adopting an NGR integrin-binding motif.^146^ The latter supports alternative attachment and/or receptor-entry using integrin β1 (ITGB1).^146^ With VR-IV adjacent to the AAVR receptor footprint (see below), one asks whether receptor “switching” involves only gain of a dominant new receptor or also ablation of AAVR interactions.^82,83^ Overlaying the AAV2–AAVR complex, VR-IV is closer to AAVR in AAVhum.8 (4.1 Å) than AAV8 (6.6 Å), but there is no incompatible clash.^83,180^ More subtle effects on solvation or flexibility are not ruled out, but, at this point, there is no evidence for AAVR interference despite the suggestive proximity. Exciting is this work’s proof of principle that structure-guided directed evolution can access new tropisms, and its illustration of cryo-EM’s value in rationalizing new phenotypes and illuminating paths to further improvement.^146^

The growing ease with which cryo-EM structures can be obtained has opened the door to comparative studies of related isolates in efforts to identify the amino acid determinants of phenotype. AAV9 from clade F (Figure 2) was reported to be more efficient than other AAVs in crossing the blood–brain barrier (BBB).^150,171,173^ Several members of clade E share this property, and with natural hosts of rhesus (rh) macaques, have the advantage of immune naivete in human (hu) patients.^181−183^ The determinants were narrowed down through the screening of AAVrh.10 and AAV1 chimera, but the biological mechanism(s) of CNS tropism have remained obscure.^184^ Several structures of rhesus and human clade E AAVs have now been determined and compared to AAV8.^135,141^ A surface region had previously been implicated in CNS tropism through analysis of chimeric serotypes^184^ but there were no significant differences in structure, focusing attention back to AAV8/9 sequence differences at S269 and N472.^135^

The availability of several related clade E structures allowed a fresh look at several questions. For three AAVs, reconstructions of DNA-containing vectors were paired with DNA-free empty particles.^135^ The genome-packing differences in capsid structure, reported previously for AAV1 at nanometer resolution,^85^ were not observed.^135^ Disordered features were observed along the 5-fold pore for all three DNA-containing vectors and AAVrh.10 empty particles. This suggests that residues, near the transition from VP1u to VP3, are passing along the 5-fold from the exterior toward the start of βA strand on the interior surface, for up to 20% of capsid proteins.^135^ This is reminiscent of autonomous parvovirus crystal structures.^14,15^ Interior basket-like features surrounding the 5-folds, seen in empty particles of AAV8 and AAVrh.39 (not AAVrh.10), could indicate alternative disordered configurations of the VP N-termini.^135^ In the DNA-containing particles, features in an interior pocket near the 3-fold were interpreted as a partially ordered 3-nucleotide fragments of single-stranded DNA.

Using AAV9 as the model system, changes upon endosomal-like acidification have been investigated at pH ranging from 7.4 to 4.^144^ There are no significant changes to the capsid protein atomic coordinates, but there are changes in disorder. Most interesting are changes in the strengths of columnar features running along the 5-fold pore, which were approximately inversely correlated with the strength of basket structures on the interior surface.^144^ These are both features that have been seen in other AAV structures (see above). Particularly intriguing was the overall strengthening and lengthening of the 5-fold pore feature as the pH is dropped. The change is not monotonic, which the authors interpret in terms of needed externalization, more specifically, of VP1u at acidic pH.^144^ Also, the authors report that the morphology of the basket features differ by pH.^144^ It is unclear how much one should read into (subtle) differences in the strength and shape of features, particularly when atomic-detail interpretation is not possible, because electron microscopy does not yet have a strong grasp on the factors that may influence signal and noise levels in individual reconstructions (see section 3.4.2). However, this does not detract from the intriguing observations that suggest increasing occupancy of the pore at low pH and increasing disorder in the immediately surrounding capsid protein as the peptide is accommodated.^144^ It seems likely that this is just the first of many studies that will use cryo-EM to examine the influence of physiological changes in environment to the AAV capsid.

AAVv66 exemplifies another opportunity brought by cryo-EM. Variant 66, isolated from a surgical specimen, differs at 13 sites from AAV2, leading to improved production, stability, and CNS transduction.^142^ The cryo-EM was just one part of a comprehensive analysis to understand the variant phenotypes. Interestingly, in spite of its close homology, the surface electrostatic potential of AAVv66, calculated from the structure, lacks the positively charged features of AAV2 because all the arginines most responsible for HSPG-binding in the clade B and C AAVs have been substituted by neutral amino acids, suggesting a different mode of cell attachment.^142^ While the detail visible varies somewhat in EM reconstructions of nominally the same resolution, this study shows that 2.5 Å can be enough to resolve many of the peptide carbonyls and side chain configuration, providing confidence in interpretation of atomic interactions.

Exemplar structures have now been pushed beyond 2 Å and resolutions attainable with crystallography. Structures of an AAV2 mutant and AAV-DJ at 1.8 and 1.6 Å, respectively, will be discussed in the technical section (section 3.2).

### 2.4 Nonprimate AAVs

Almost all AAV structural studies have been with primate viruses. This is beginning to change with the observation that it is often possible to cross-package an AAV2-based vector DNA in a capsid to which humans will be immune-naïve, perhaps offering attractive traits. BtAAV-10HB, isolated from bats, is the first structure of a nonprimate AAV^143^ DNA-containing and empty particle sets of BtAAV-10HB were separated on 2D classification, and the resulting atomic structures were compared to AAV2 and AAV5, the most divergent representatives of prior structures (Figure 2).^1,185^ The core of the bat virus structure differs little from AAV2 and AAV5: rmsd values of 0.6 Å are not experimentally significant at this resolution. The differences are in the surface loop variable regions, particularly VR-I, VR-III, and VR-VII, where BtAAV-10HB differs by >4 Å *vs* AAV2 and/or AAV5. The authors suggest that the absence of surface differences in 5-fold proximal loops might reflect conservation of the region proposed as important in Rep protein interaction, DNA packaging, and extrusion of VP1u.^143^ Like other recent structures (see above), there are disordered map features consistent with the N-termini of a fraction of capsid proteins starting on the exterior and connecting to the β-barrel through the 5-fold pore.^143^

### 2.5 Complexes with Antibodies

An understanding of AAV’s immune interactions is not just of fundamental interest but is a priority in the development of gene therapies. Most in the human population have been exposed naturally to AAV and are seropositive to one or more serotypes.^89,186−188^ Circulating neutralizing antibodies (NAb) diminish transduction efficiency following systemic injection, limiting eligibility for ongoing clinical trials and approved treatments.^62,189−196^ Additionally, because transduction is usually episomal, many therapies will have finite longevities, depending on delivered copy number, cellular turnover of targeted cells, and expression needed for therapeutic effect.^197,198^ Readministration is considered a likely future requirement.^199−204^ Strategies for mitigating vector neutralization, resulting from prior natural or vector exposure, could include immune suppression (to be avoided), use of vectors based on diverse natural serotypes, or vectors modified by rational design or directed evolution for immune escape.^166,200,201,205−219^ Structural biology can provide insights into mechanisms of neutralization and a roadmap with which to plan capsid modifications. It might also help in understanding distinctions between binding and neutralizing antibodies. Intuitively, one might expect this to be important in the immune screening of candidate gene therapy patients, but note that antibody-mediated transduction inhibition in mice was not specifically dependent upon neutralizing antibodies.^196,220^

Cryo-EM has long been favored over X-ray crystallography for structure of antibody complexes because of the difficulty of achieving uniform binding of antibodies to 60 symmetry-equivalent antigenic sites, then crystallizing such a large complex. Cryo-EM structures have been determined for several of the better characterized serotypes in complex with fragments of monoclonal antibodies from hybridomas screened usually previously. Much of this work was performed prior to the “cryo-EM revolution”, and therefore at nanometer resolutions. Particularly exciting is the publication of two structures in 2018 (Table 2) at 3–4 Å resolution, showing that limits had been instrumentation, not sample. The complexes have provided excellent insights into immune recognition and neutralization, but it is a disappointment that the full benefit of multiple antibody studies has not been realized through deposition of structures and maps in public databases. Such deposition is the prevailing expectation in the structural biology community and a requirement for publication in most journals. Authors may have interests in intellectual property, but the situation is exacerbated by publication in virological and gene therapy journals where the roles of reviewers and editors in ensuring access to published structural data has apparently not been understood.

**Table 2:** Cryo-EM Studies of AAV Complexes with Fab Fragments of Monoclonal Antibodies

serotype	mAb	neutralizing	PDB	EMDB	resolution (Å)	capsid	date	ref
AAV2	A20	yes	3J1S	5424	6.7^a^	wtAAV	2012	(167)
AAV2	C37B	yes			11	VLP	2013	(221)
AAV1	ADK1a	yes			11	VLP	2015	(222)
AAV1	ADK1b	yes			11	VLP	2015	(222)
AAV1	4E4	yes			12	VLP	2013	(221)
AAV1	5H7	yes			23	VLP	2013	(221)
AAV6	5H7	yes			15	VLP	2013	(221)
AAV6	ADK6	yes			16	VLP	2018	(223)
AAV5	ADK5a	?			11	VLP	2015	(222)
AAV5	ADK5b	yes			12	VLP	2015	(222)
AAV5	3C5	no?			15	VLP	2013	(221)
AAV5	HL2476	yes			3.1	VLP	2018	(167,224)
AAV8	ADK8	yes			19	VLP	2012	(225)
AAV9	PAV9.1	yes			4.2	vector	2018	(222,226)

a In 2009, AAV2:A20 was annotated as FSC_0.5_ = 8.5 Å, but currently it is more prevalent to quote FSC_0.143_ = 6.7 Å as the resolution.

#### 2.5.1 Antibody Complexes at High Resolution

Structures of complexes with newly generated antibodies are at higher resolution than previously attainable. The complementarity determining regions (CDRs) of PAV9.1 interact with the exterior tips of the VR-V and VR-VIII loops from adjacent subunits where they come together on the 3-fold-facing surface of the spike.^226^ Gln_590_ from the VR-VIII antigenic region had been implicated, through mutagenesis, in transduction.^227^ Given sequence alignment with the HBD of AAV2, Pulicherla *et al.* had hypothesized that PAV9.1 was blocking receptor-binding, what we would now call glycan attachment.^227^ The subsequently determined galactose-binding site is in the same general region but not in close proximity.^144^ Now, in retrospect, we see overlap between the PAV9.1 epitope and the footprint of AAVR when AAV9 and AAV2 are superimposed.^83,228,229^ Indeed, the side chain of AAV9 Gln_590_ appears to interact with the backbone of AAVR PKD2 if the AAV2–AAVR complex is superimposed.^83^ This suggests that the antibody might be neutralizing by competing with AAVR-binding. However, it might not be so straightforward. A second AAV9 mutation (W503R) in the PAV9.1 epitope is closer to the galactose site and has been implicated in both decreased glycan binding and liver detargeting, thought to be mediated by interference with glycan-associated liver sequestration.^230^ The mechanisms of PAV9.1 neutralization might be quite complex because AAV9 W503 is also in contact with superimposed AAVR–PAV9.1,which might interfere with both glycan attachment and protein receptor binding at overlapping sites. PAV9.1 is the first AAV9 monoclonal to be characterized. In binding to the 3-fold spike, it resembles some antibodies to other serotypes (see below). PAV9.1 is unusual in that it binds to the 3-fold proximal inner surface of the spike, such that only one Fab could bind per trimer. High neutralizing activity is attained even though saturation would require binding at 20 of the 60 symmetry-related sites.^226^ AAV9 mutations designed to disrupt the visualized interface had a PAV9.1 neutralization escape phenotype (validating the structure) but did not confer binding escape to neutralizing polyclonal sera from mice, macaques, or humans, suggesting that the epitope is not dominant and is one of many targeted by diverse paratopes *in vivo*.^226^ From the available structures, our understanding is incomplete. The close proximity of different phenotypic determinants in AAV9 highlights the challenge of designing immune-evading vectors without affecting cell entry.

The highest resolution AAV-antibody complex (3.1 Å; AAV5:HL2476) permitted *de novo* atomic modeling, as opposed to the “pseudo-atomic” usually rigid docking of a homology model into lower resolution maps.^224^ HL2476 recognizes an epitope on the 3-fold spikes of AAV5 that is quite analogous to the PAV9.1 epitope on AAV9. It comes within 6 Å of amino acids reported to form the AAV5 sialic acid pocket A at the depression on the 3-fold axis but is distant from the sialic acid B site at the 5-fold-facing base of the spike.^231,232^ Thus, Jose *et al.* concluded that neutralization was through partial occlusion of glycan primary receptor (now termed attachment factor).^224^ It is noted that the AAV5 residues contacting HL2476 are immediately adjacent to the PKD1 footprint of receptor AAVR (see below).^147^ If coordinates were available, it seems certain that there would be conflict of the Fab arm with AAVR, while it is also plausible that the Fab heavy chain could obstruct access of a polysaccharide chain to the terminal SIA site A on the 3-fold.^147,224,232,233^ So, the evidence is less direct, but HL2476 might interfere with AAVR receptor-binding, and possibly glycan attachment, like PAV9.1.^224^ As to be expected, the map for the “ligand” Fab is somewhat less ordered than within the AAV5 capsid, with the maps clearly defining the aromatic CDR side chains, but smaller and flexible side chains are not as well defined as in the AAV5 VR-IV and VR-VIII loops with which they interact.^224^ Ambiguities depend both on overall resolution and local disorder. At the interfaces of interest, in this the highest resolution AAV:Fab complex at 3.1 Å, the identities of amino acids that are neighbors are in no doubt, while assignment of individual atomic interactions, like hydrogen bonds, will be of mixed reliability.

### 2.6 AAV–Antibody Complexes at Intermediate and Low Resolution

Aside from the recent HL2476 and PAV9.1 complexes, the highest resolution (6.7 Å) came from the first of the studies, a complex of AAV2 with the strongly neutralizing monoclonal A20 (Table 2).^167,234^ This structure was long in gestation with a challenge that, fortunately, has not been seen again. By way of background, cryo-EM requires superposition of thousands of particles, so it is common to work with the “business” domains, absent other parts that might be flexible and detract from precise alignment. Thus, structures of virus–antibody complexes are most commonly pursued with monovalent Fab fragments, prepared by papain digestion.^235−237^ Unexpectedly, A20 Fab fragments did not compete with intact mAb in competition enzyme-linked immunosorbent assay (ELISA) and proved to be unstable, particularly at low temperature. Functional binding was only achieved with fragments prepared by pepsin digestion, then separation of Fab’ arms under reducing conditions.^167^

**Figure 8:**
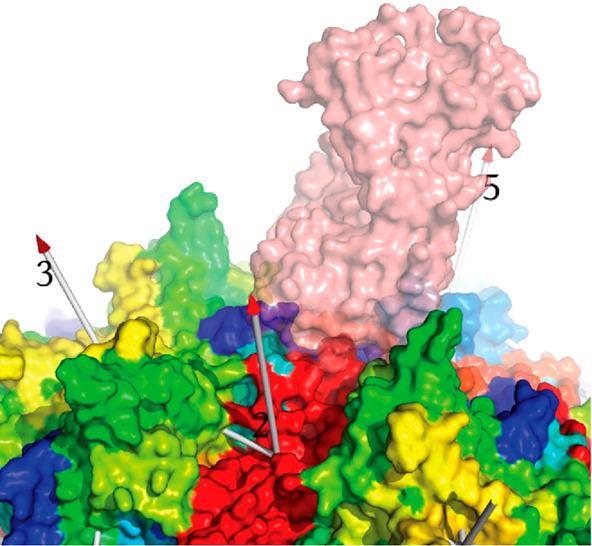
Epitope mapping by cryo-EM. Although not of atomic resolution, the reconstruction was clear enough to dock a canonical Fab′ arm (salmon). The capsid structure is rainbow colored from blue (Gly_217_) to red (Leu_735_), so that contributions of different variable regions (VR) to the epitope can be discerned. Epitope residues are in dimmed color, looking through the lower end of the translucently rendered Fab′A20. The principal contributions to the epitope are: (a) Ser_261_–Ser_264_ of VR-I, together with Asn_253_, Asn_254_, and Lys_258_ of the canyon/wall that are colored red. (b) Ser_384_–Gln_385_ of VR-III (aquamarine). (c) From a 2nd subunit, Val_708_ of VR IX and Asn_717_ (red). (d) Glu_548_ and Lys_556_ from VR-VII (chartreuse) and on an adjacent 3-fold spike and Ser_658_–Thr_660_ (brown) in the canyon from a 3rd subunit. The epitope is clearly conformational, including several peptide segments and three subunits, so specific for assembled complexes. The AAV2-Fab′A20 complex shown is the only antibody complex available on public databases, but other structural mappings of epitopes followed a similar process of docking a “pseudo-atomic” model into usually low-resolution cryo-EM of an antibody complex to identify AAV contact amino acids.^167^

The A20 consensus (core) footprint stretches from a plateau on the side of each 3-fold spike down toward the canyon that encircles the 5-fold axis (Figure 8).^167^ It includes residues from hypervariable surface loops VR-I, -III, and -IX, the latter from a 3-fold related neighboring subunit, thereby rationalizing the prior observations that A20 is a conformational epitope that recognizes only assembled particles.^234,238^ A20 had previously been subject to epitope mapping through mutagenesis and peptide scanning.^238−242^ Of sites that remained plausible after the AAV2 structure, all are in the visualized footprint, which also extends into the more conserved canyon that had not previously been probed.^1,167^ There is no overlap between A20 and the HSPG attachment site (see below), consistent with its inability to inhibit cell binding.^238,243^ A20 is widely regarded as inhibiting a post (cell) entry step and is sometimes stated as inhibiting a post nuclear entry step.^210,221,222^ However, the primary citation presents nothing more than evidence for uninhibited cell attachment in the presence of A20.^238^ It seems likely that confusion resulted from the naming of proteoglycans as receptors, often implicitly associated with entry, rather than as attachment factors (see introductory discussion).^105^ Postentry neutralization was given added credence when the AAVR receptor site was reported to be distinct from the A20 epitope.^82^ However, this analysis appears incorrect, at odds with the report of Meyer *et al.* that A20 and AAVR overlap when cryo-EM structures of their complexes are overlaid.^83^ The latter suggests neutralization mediated by competition with AAVR-receptor binding.^233^ Later, there would be the intriguing observation that the most distinctive feature in the structure of the AAV-DJ vector was the unique conformation of VR-I within the epitope, explaining the vector’s escape from A20 and suggesting that the main driver in AAV-DJ’s directed evolution might have been escape from hypothetical A20-like antibodies that could have been within the pooled polyclonal human serum used to select immune resistance.^132,166^

The AAV2:A20 structure was at 6.7 Å resolution, which, as explained in section 3.3.1, allows atomic modeling and mapping of an epitope boundary with ∼2 Å accuracy, or slightly better than ±1 amino acid. Structures below range from 11–23 Å resolution, so we should expect errors in defining the epitope boundary to be the width of one or two amino acids. The general vicinity of binding (*e.g*. spike *vs* plateau) should be robustly determined. However, the experimental errors are large enough that they could sometimes affect assessment of overlap with cell attachment and receptor sites or other functional sites that could be important for mechanisms of neutralization. It is important that the interpretation of each structure be in the context of understanding the likely errors over widely differing resolutions (section 3.3.1).

**Figure 9:**
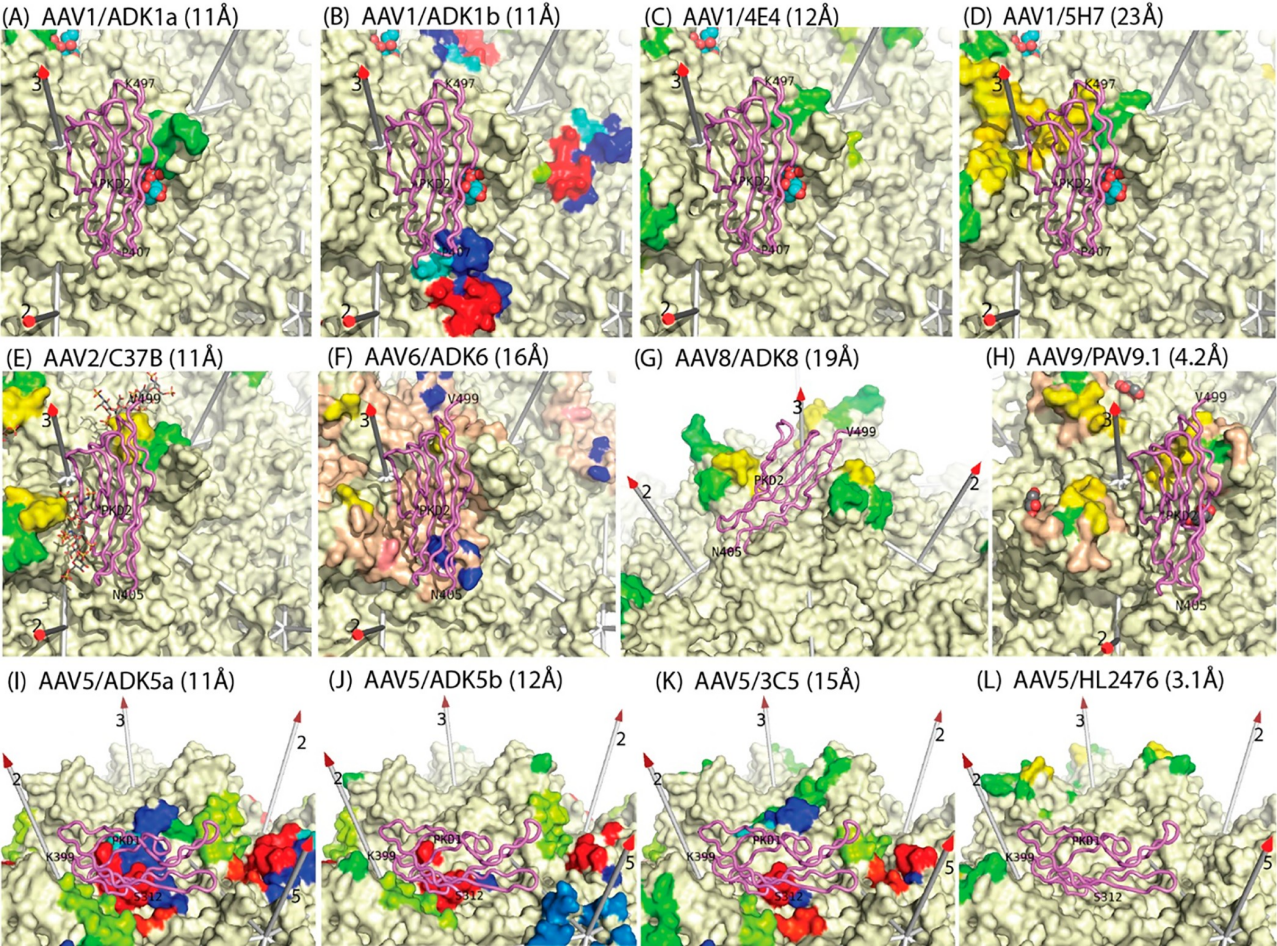
Epitopes of additional anti-AAV monoclonal antibodies. In addition to the pseudoatomic model coordinates available for the AAV2–Fab′A20 complex (Figure 8^167^), lists of contact residues have been reported, derived from cryo-EM studies of 12 other Fabs at resolutions noted parenthetically.^221−226^ Contact residues are highlighted on the surfaces of the respective serotypes,^1,13,81,137,147,150^ rainbow-colored by residue number (blue to red) to distinguish different VRs. The rest of the surfaces are cream colored, except for the wheat-colored regions in F and G, reported as occluded by antibody binding.^223,226^ For AAV1 and AAV2, known glycan attachment sites are indicated with the overlaid sialic acid (spheres) or fondaparinux (stick-model), respectively.^244,245^ Glycan attachment has not been observed directly for other serotypes, but mutational analysis for dual-binding AAV6 indicates a sialic acid site with AAV1 and heparan-binding like AAV2 with participation of Lys_531_ (salmon-colored).^81,245^ Bound domains from the subsequent cryo-EM complexes of AAVR are also overlaid with violet backbone traces: PKD1 for AAV5 and PKD2 for AAV1 and AAV2 (with AAV6–9 assumed to be similar).^83,137,147,246,247^ Comparative analysis tells us that: (1) dominant antigenic regions include the spikes (tip and sides) and the spur that runs toward the 2-fold.^248^ (2) Many, but not all neutralizing antibodies occlude glycan attachment. (3) The binding of all neutralizing antibodies conflicts with the binding of the serotype-relevant AAVR domain. In most cases, PKD1 or PKD2 lies directly over the neutralizing epitope. For HL2476 (L) conflict is with the implied location of the unseen interdomain linker (several others show direct conflict with PKD2 as well as implied conflict with the linker, as illustrated for AAV2/A20 in Figure 12).

Eleven structures of AAV complexes with monoclonal Fabs followed that of A20, mostly from the group of Mavis Agbandje-McKenna. They are at somewhat lower resolution but key for comparative analysis (Figure 9).^210,221−223,225^ First was a cryo-EM reconstruction of the AAV8:ADK8 complex at 18.7 Å resolution, which, in the absence of a protein sequence, was fit with the structure of a generic (unrelated) Fab.^225^ At this resolution, ADK8’s footprint appears to include all of the exposed loops near the top of the 3-fold spike.^225^ The atomic model was an approximation but was the foundation for structure-directed mutagenesis, with penta- and hexapeptide peptide replacements from AAV2 in VR-IV, -V, and -VIII. VR-IV, and -V had no impact upon ADK8-binding as measured by ELISA, but binding was abrogated by multiple mutations within VR-VIII, which is the loop of the spike on the 3-fold proximal surface.^225^ ADK8 preincubation significantly reduced cytoplasmic presence and abrogated *peri*-nuclear accumulation but did not affect cell attachment.^225^ Thus, it is considered to act by inhibition of cell entry and/or trafficking.^225^

Next were complexes of three newly isolated monoclonal Fab with four AAV serotypes.^221,249^ Approaches were similar, except that Fab homology modeling was by the WAM algorithm when sequences were available (see section 3.3.1.2).^250^ mAb 5H7 is cross-reactive for the closely related serotypes AAV1 and AAV7. Fab 5H7 binds between two of three spikes surrounding each 3-fold axis, with steric occlusion limiting Fab-binding to one per 3-fold (Figure 9).^221^ C37-B and 3C5 fully saturate at one Fab per capsid subunit, while 4E4 would overlap with a 2-fold related neighbor if both sites were occupied.^221^ 5H7, C37-B, and 4E4 are neutralizing antibodies and bind to the most exposed regions near the top or sides of the 3-fold spikes (Figure 9).^221,238,249^ The authors reported general proximity to the binding sites of glycan attachment factors (then termed receptors). In the case of the AAV2:C37-B complex, the epitope includes Arg_585_ and Arg_588_ of the HBD in VR-VIII, exactly where HS analogues had been located in structures described below.^243,244^ Thus the C37-B complex structure explains its observed inhibition of AAV2 cellular attachment.^221,238^ Now that structures of the AAV2–AAVR complex are known (see below), overlay indicates some direct overlap between many of the epitopes and the receptor footprint (Figure 9).^82,83,233^ By comparing to the subsequent AAV2–AAVR cryo-electron tomography (see below), we see that a single 5H7 or C-37B Fab bound per 3-fold, extending radially from the virus, would conflict with the AAVR PKD3 domain similarly extending away from any of the three symmetry-related PKD2 binding sites.^83,221^ An analogous rationalization could explain the relatively high neutralizing activity of 4E4.^249^ Its one-of-two site occupancy of leaves open surface sites for the binding of AAVR PKD2. Gurda *et al.* note extension of Fab 4E4 to the 2-fold axis, where the unseen PKD1 domain of the AAV2–AAVR complex would have to be, so above-surface competition is a possible explanation. Quite different is the anti-AAV5 3C5, which is categorized as a non-neutralizing Fab by Gurda *et al.*(221) With the *caveat* of Gurda *et al.*, that the map is fragmented and interpretation ambiguous,^221^ the Fab is oriented with CDRs interacting with the more conserved canyon region encircling the 5-fold. In an unusual disposition, Fab 3C5 is modeled tangential to the AAV surface, with the constant region passing over the plateau, near an AAV5 sialic acid attachment site (see below) and toward the 3-fold spike.^221,232^ The CDR-recognized epitope lies directly underneath the PKD1 domain in the AAVR complex with AAV5 (Figure 9).^137,147,221^ It is difficult to imagine that there would not be conflict. Indeed, the primary citation indicates weak neutralization by the intact IgG, albeit 10-fold weaker than 5H7 and 100-fold weaker than 4E4, so a hypothetically weaker 3C5-binding could mean that a higher concentration would be needed to see the any effects upon viral transduction of the antibody-receptor overlap apparent through structure.^147,221,249^

Another comparative study from the Agbandje-McKenna group looked at four monoclonals, two against AAV1 and two against AAV5.^222^ Illustrative of approximations in nanometer resolution epitopes, *pseudo*-atomic modeling gave an initial ADK1a contact list of eight residues and 260 Å^2^ in area, well below typical antigen–antibody interfaces, so more generous footprints were obtained by visual inspection of cryo-EM maps overlaid on the AAV atomic coordinates.^251,252^ For these, the accuracy is commensurate with the nanometer resolution, or ±3 amino acid positions. Such accuracy is plenty for the general conclusions to be drawn. ADK1a and ADK1b are, like 4E4 and 5H7, anti-AAV1 neutralizing monoclonals.^221,249^ With a single exception, their binding sites are near the top and sides of the 3-fold proximal spikes, just like the antibody against the closely related AAV2, C37-B (Figure 9). The exception is ADK1b which is described as binding to the 2/5-fold wall, a region referred to by others as the plateau, and there is much overlap with the footprint of AAV2’s A20.^167,222^ ADK1b contact residues include those that impact muscle tropism and transduction efficiency, but categorization as having a postentry neutralization mechanism seems to be based upon correspondence of the ADK1b and A20 footprints and so should similarly be open to reappraisal, given overlap between the ADK1b epitope and the binding site of AAVR on AAV1.^137,168^ The ADK1b epitope comes within about 7 Å of the bound SIA, so interference with an attached polysaccharide cannot be ruled out, but, now superimposing the observed bound structure of AAVR PKD2,^137^ it is clear that conflict with PKD1 and the inferred location of the PKD1–PKD2 linker eliminates the possibility of binding AAVR at sites where ADK1b is bound (Figure 9). For AKD1a, the mechanism of neutralization was proposed to be inhibition of glycan attachment, due to the juxtaposition of the SIA binding site to the epitope and escape from ADK1a recognition by nonbinding AAV1 variants.^245^ Now with the structure of an AAVR complex,^137^ we see the colocalization of receptor, glycan, and AKD1a epitope (Figure 9): it is difficult to imagine how AKD1a could not be interfering with both glycan attachment and receptor-binding. 4E4 and 5H7 inhibit cell attachment, and with epitopes on the spike, it might be natural to assume similar glycan exclusion as in ADK1a, but actually, the epitopes are on different sides of the spike, leaving the SIA site sheltered (Figure 9^221,249^), at least from direct conflict with the CDRs. In both cases, there is greater overlap of epitopes with bound PKD2 of AAVR (Figure 9). The overlap is substantial for 5H7, perhaps accounting for high neutralizing activity of 5H7 as a Fab,^249^ whereas 4E4, observed to be more neutralizing as an intact antibody,^249^ would conflict more if symmetry-related epitopes are bivalently bridged through the PKD2 site (Figure 9).

The contact sites for ADK5a and ADK5b have considerable overlap with the overlaid contacts of 3C5 (on AAV5).^167,221,222^ The core region of interaction, shared by all, is in the 5-fold encircling canyon, immediately underneath and surely in conflict with the bound PKD1 domain of AAVR as it bridges toward the AAV 5-fold axis.^137,147,222^ Why then should ADK5b be neutralizing, but ADK5a and 3C5 not be? Above, it has been noted that 3C5 is actually weakly neutralizing. Similarly, ADK5a is not completely non-neutralizing, but 100-fold more weakly neutralizing than ADK5b.^222,249^ When tested at 100-fold higher concentration, ADK5a, like ADK5b, competed in AAV5-binding with AAVR PKD1.^147^

In summary, cryo-EM has allowed identification of antigenic regions, but, on recent integration with analyses of glycan-attachment and receptor-binding, assumptions about neutralization mechanism are called into question. Much remains to be learned. It is noted that progress has come through structures of complexes, some of which are at modest nanometer resolution, but this can be sufficient to constrain the interpretation of virological and immunological data.

### 2.7 Complexes with Glycan Cellular Attachment Factors

Readers are reminded that the glycans that mediate AAV attachment have historically been known as (primary) receptors, and it may take some time for the more appropriate term “attachment factor” to take full hold in the literature.^105,233^ The preferred glycan varies by serotype, as usually determined by transduction assay, either modulating cell surface glycan expression or as inhibited by the presence of specific glycans.^107,253^ The glycans are mostly negatively charged: heparan sulfate (HS) for AAV2, 3B, 6, and 13,^105,109,110,254^ and sialic acid (SIA) for AAV1, 5, and 6 (α2–3 and α2–6 N-linked) and AAV4 (α2–3 O-linked),^106−108^ with galactose as an uncharged exception (AAV9).^255^

Glycan adducts were the first AAV complexes subject to cryo-EM (Table 3). This was of necessity, as the addition of heparin fragments was disruptive to AAV2 crystals. In 2009, long before the “EM resolution revolution”,^256^ two concurrent studies came to two very different conclusions. At 8 Å resolution, O’Donnell *et al.* found fragments of a heparin polymer wrapped around each 3-fold spike, binding directly to Arg_585_ and Arg_588_ that mutagenesis studies had implicated, among others, in heparin-binding.^243,257^ By contrast, Levy *et al.* found 0.5 to 1σ features in a difference map (sections 3.3.1.1 and 3.4.2) at 18 Å resolution that suggested: (a) heparin-binding remote from the arginines and (b) capsid conformational changes that hinted at a dynamic process whereby heparin and arginines might be brought into proximity. It was a captivating and widely cited possibility that glycan binding might be triggering conformational changes, needed eventually to release VP1u and DNA from inside the capsid. However, it was the more mundane O’Donnell *et al.* results that would be sustained by subsequent higher resolution studies (below).^244,258^

**Table 3:** AAV Complexes with Glycan Attachment Factor Analogues

serotype	ligand	PDB	EMDB	resolution (Å)	capsid	date	ref
*AAV2*	heparin			8.3	wt	2009	(243)
*AAV2*	heparin			18.0	VLP	2009	(259)
*AAV-DJ*	sucrose octasulfate (SOS)	3J4P	5681	4.8	VLP	2013	(258)
*AAV-DJ*	fondaparinux	5UF6	8574	2.8	VLP	2017	(244)
*AAV9*	galactose	7MTZ	24000	2.7	VLP	2021	pH 7.4 ^144^
*AAV9*	galactose	7MUA	24003	2.4	VLP	2021	pH 5.5 ^144^

It is a matter of speculation how the results of two similar studies could be so different. In terms of sample preparation, both groups tried premixing AAV2 and heparin, but O’Donnell *et al.* switched to preadhering AAV to EM grids, because, in free solution, the mixture aggregated with little left monodisperse for EM viewing.^244^ It is possible that a nonaggregated fraction of AAV would be low in heparin binding and that this is what was imaged by Levy *et al*. The challenges of difference maps and the particular dangers at low resolution are discussed in section 3.3.1.1. Resolving the conflict would require higher resolution and use of pure synthetic glycan analogues rather than the heterogeneous mixture of sequences in natural heparin preparations. The use of synthetic analogues introduces two caveats for the work summarized below. First, the functional groups might differ and so too their interactions. Second, synthesis of glycan analogues is limited to short oligomers, so the features of any multisite binding are lost. At the time, resolution of the chemical groups of a ligand by cryo-EM was an extremely ambitious goal.

Success came first with a complex of AAV-DJ and sucrose octasulfate (SOS) at 4.8 Å resolution.^258^ We do not expect to see the details of protein structure at 4.8 Å, but most of the sulfate groups proved to be discernible, even with ambiguity between the glucose and fructose rings of the SOS. In analysis, attention to detail was needed. Difference maps were calculated following correction of a 1% error in magnification and scaling of the “density” values of the native and complex maps, both with reference to the atomic model of the virus.^261^ SOS appeared not to be bound at all sites, thus, the protein structure visualized was an averaged mixture of bound and unbound forms. For improved clarity on conformational changes, the ligand occupancy was refined, and then an occupancy-weighted difference map was calculated, designed to subtract an unbound map component, leaving only an image of the complex.^261,262^ At 4.8 Å resolution, flexible atomic refinement is marginal.^261^ With additional restraints on torsion angles, all-atom refinement led to rmsd changes of 1.8 Å, but many of the largest changes were in loops of poor quality in the difference map. The one exception was residues 584–589, where clear density showed modestly displaced conformation. This region was added (with the SOS ligand) as a second conformer (with an occupancy that optimized to 35%) for refinement of a native/bound mixture against the reconstruction of the complex. An upper-bound estimate of coordinate error (±1.4 Å) came from a comparison to the high-resolution crystal structures of the parent serotypes of the AAV-DJ chimera. Side chains are not well resolved at 4.8 Å resolution, but Arg_590_ of the HBD can interact without moving, while an observed 1 Å displacement of the top of the VR-VIII loop requires a 4.7 Å shift to keep Arg_587_ within the map envelope and interacting with another SOS sulfate. A neighboring loop (484–9) has displacements of up to 2.1 Å, but elsewhere, refinements against native and complex reconstructions differ by 0.9 Å, insignificant at 4.8 Å resolution. Thus, this was confirmation that ligand-induced changes were local to the glycan binding site.

**Figure 10:**
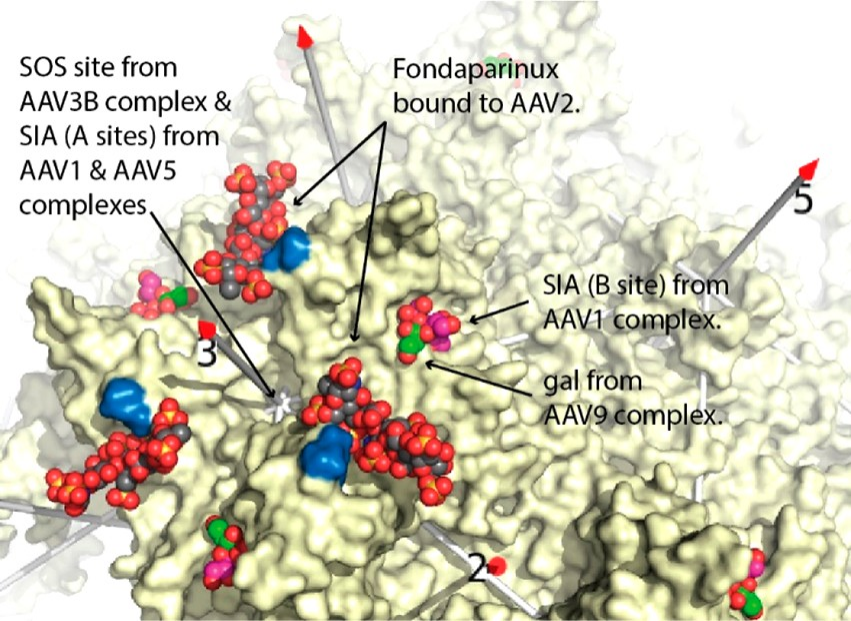
Comparison of glycan attachment sites overlaid on the structure of AAV2. Shown are fondaparinux, a heparin analogue (space-filling, gray carbons) from the AAV2 cryo-EM complex,^244^ sialic acid (SIA, magenta carbons) from the AAV1 crystal structure,^245^ and galactose (gal; green carbons) from the cryo-EM AAV9 structure,^144^ the latter two overlapping. Fondaparinux is attaching at arginines 585 and 588 (blue) of the heparin-binding domain on the surface of AAV2.^257^ Atomic models are not available, but additional glycan attachment interactions for AAV1, AAV3B, and AAV5 have also been localized through crystallography and/or mutation to the surface at the 3-fold axis.^232,245,260^ (A second AAV5-SIA site was buried where the HI loop (orange in Figure 4) disappears under the 5-fold side of the spike, but it is likely not functionally relevant because mutation leads to SIA-independent change in transduction.^232^) The fondaparinux heparan analogue and SIA/galactose are attached on opposite sides of each 3-fold proximal spike. Actually, they are closer with a 3-fold rotation that brings the sites to opposite sides of a valley running from 2-fold to 3-fold, which may be relevant to protein receptor-binding.

More definitive was a subsequent AAV-DJ:fondaparinux complex, which, like the SOS complex, confirmed binding at the heparin site of O’Donnell *et al.* (Figure 10). Fondaparanux is a pentasaccharide pharmaceutical, about the longest synthetic heparin analogue available. In a difference map, the fondaparinux (Arixtra) is by far the highest peak (13σ), but this was limited in resolution by the 4.5 Å native data set at the time.^244^ Thus, a dual conformer model (bound + native) was refined into the reconstruction of the complex, yielding a bound-form occupancy of ∼0.33. The low occupancy is consistent with surface plasmon resonance (SPR), indicating weaker binding than heparin (*K*_D_ ≈ 10 mM).^244^ Detailed features of fondaparinux were not apparent in the map, indicative of disorder in binding configurations. It does interact with the implicated arginines. The map for the virus is clear, including most side chains, and there is no evidence of widespread conformational changes on fondaparinux binding, either in displaced atomic model or in difference map peaks that one would expect to see. By comparing the structure of this complex to native AAV structures, variation in the arginine side chains suggested that different glycan sequences could be accommodated with local adaptation, providing a rationale for an observed lower glycan specificity than had been anticipated.^95,263^

As complexes with HSPG analogues were pursued at the improving resolutions attainable with cryo-EM, other glycan–AAV complexes could be pursued by crystallography, including AAV3 with SOS and AAV1 or AAV5 with sialic acid (Figure 10).^232,260,264^ At this point, cryo-EM is easier and just as capable of high resolution. As part of the study of pH-induced transitions in AAV9, complexes were obtained with galactose, the terminal residue of AAV9’s attachment glycan.^144^ Although of poor definition, a feature at ∼4σ of appropriate size is seen at both neutral and acidic pH, and this feature is coordinated by five residues previously implicated in galactose binding by mutagenesis (Figure 10).^265^ Binding of galactose decreases disorder in the DE and VR-I loops, there are differences in the appearance of features along the 5-fold pore, and in the presence of a basket feature on the interior 5-fold surface at acidic pH.^144^ While care is needed in quantitative interpretation of disordered features in cryo-EM maps, the differences are interpreted as suggestions of glycan attachment affecting capsid dynamics, the nature of which are not entirely clear at this point.^144^

### 2.8 Complexes with Cell Receptors

Particularly exciting over the last two years has been the publication of cryo-EM structures of AAVs complexed with fragments of the AAVR membrane protein receptor that is essential for most serotypes. A breakthrough resulted from failed attempts at structural studies of previously reported coreceptors and even to obtain evidence of association with expressed proteins implicated by viral overlay and other methods.^109,111,113,115−118,266−268^ Fear that the wrong proteins were being targeted for structure led to the unbiased genome-wide screening for cellular host factors that were essential for AAV transduction. From this emerged the hitherto uncharacterized KIAA0319L (aka AAVR) as a *bona fide* protein receptor for viral endocytosis and trafficking toward the nucleus.^119^

AAVR is anchored to the membrane by a single transmembrane region near the C-terminus. Starting from the N-terminus, its ectodomain has a signal peptide, a MANEC domain (motif at N-terminus with eight cysteines) then five Ig-like PKD (polycystic kidney disease) domains.^269,270^ Heterologous overexpression of ectodomain fusion constructs allowed SPR measurements of AAV2-binding (*K*_D_ of ∼150 nM), the first time that strong physical interactions had been reported for a purported AAV receptor, while transduction of knockout mice with a luminescence reporter vector confirmed its significance *in vivo*.^119^ Expression of PKD-deletion mutants *AAVR-*knockout cells localized entry determinants within the PKD domains 1–3 as were the AAV2 binding sites assayed by ELISA.^119^ The binding phenotype was further localized through genetic complementation, viral overlay assay and transduction inhibition using domain-deletion mutants and domain expression constructs.^246^ This study also provided the first indications of differences among serotypes, with AAV5 dependent exclusively on PKD1, while in AAV2 both PKD1 and PKD2 affected binding with PKD2 dominant.^246^ More exhaustive analysis of serotypes showed that infection was AAVR-independent for an AAV4-like group of viruses but that AAVR was important throughout the rest of the family.^247^

Structures were pursued by the Chapman and Stagg groups in the U.S., and a collaboration between Wei Ding, Zhiyong Lou, and Zihe Rao centered at Tsinghua University. Both started with PKD1–5 constructs from the ectodomain, ending, through different paths, with high-resolution structure for just the PKD2 domain that is tightly bound (Table 4). The Chapman group used a bacterially expressed N-terminal fusion with maltose-binding protein (MBP), a construct already known to have high affinity.^119^ The Tsinghua team expressed a similar region containing PKD1–5 but with a C-terminal His-tag and without the MBP.^82^ By 2018, the Chapman group had presented intermediate resolution structure but had abandoned the fusion construct in favor of a divide-and-conquer strategy toward high resolution using a smaller PKD1–2 construct.^271^ Without the MBP domain, the Tsinghua team obtained a reconstruction at 2.8 Å for PKD1–5 in which an atomic model for the PKD2 domain could be built.^82^ These efforts benefitted either from the new THUNDER particle filtering algorithm and/or higher ligand saturation or order, absent the MBP fusion domain.^272^ In the Chapman group, cryo-EM single particle analysis of the MBP-PKD1–5 fusion had stalled at 10 Å resolution, even with subvolume classification around each 3-fold. This was used in an attempt to distinguish occupied/unoccupied sites as well as differing conformations of AAVR domains that were not interacting directly with AAV2.^83,273^ Eliminating further disorder by focusing on a PKD1–2 construct, the Chapman group attained 2.4 Å resolution but needed to confirm that the short construct represented the physiologically relevant interactions of the intact receptor.^83^ A hybrid methods approach was required, with integration of detail from the two-domain complex with low-resolution context and validation coming from cryo-electron tomography and cross-linking mass spectrometry (XL-MS or x-MS) using a more native-like 5-domain receptor complex.^274^

**Table 4:** AAV Complexes with Receptor Domains

serotype	receptor (fragment)	date	PDB	EMDB	method	resolution (Å)	source	ref
*AAV2*	AAVR (PKD1–5)	2019		0621–0624	cryo-ET	10–20	VLP	(83)
*AAV2*	AAVR (PKD1–5; see only PKD2)	2019	6IHB	9672	SPA	2.84	rAAV	(82)
*AAV2*	AAVR (PKD1–2; see only PKD2)	2019	6NZ0	0553	SPA	2.39	VLP	(83)
*AAV1*	AAVR (PKD1–5; see only PKD2)	2019	6JCQ	9794	SPA	3.30	rAAV	(137)
*AAV5*	AAVR (PKD1–5; see only PKD1)	2019	6JCS	9796	SPA	3.18	rAAV	(137)
*AAV5*	AAVR (PKD1–5; see only PKD1)	2020	7KPN	22988	SPA	2.51	VLP	(147)

#### 2.8.1 Big Picture: Cryo-electron Tomography

Cryogenic electron tomography (cryo-ET) has recently been applied to AAV to characterize the substantial heterogeneity in AAVR complexes. Cryo-ET differs from conventional (SPA) cryo-EM in that each particle is imaged from multiple directions, providing 3D information for every particle which is advantageous when classifying and averaging heterogeneous elements.^275,276^ The tradeoff is spread of the possible electron beam dose over multiple images of lower signal and therefore lower resolution than SPA. Further technical details of the approach are provided in section 3.5. Cryo-ET had substantial impact in the characterization of AAVR complexes that exhibited two types of heterogeneity: (a) the MBP-PKD1–5 construct was bound at only an asymmetric fraction of the 60 symmetry-equivalent sites on AAV2, likely due to steric occlusion, and (b) AAVR has multiple domains joined by flexible linkers with heterogeneity in the disposition of domains not interacting directly with AAV. Cryo-ET became the anchor of a holistic approach, providing overall configuration and context for detailed SPA analysis of the interactions of individual domains to be described in section 2.8.2.

The preferred overall conformations of AAV2/AAVR complexes were revealed by cryo-ET with subvolume classification and averaging. The raw tomograms provided compelling visual evidence of a stable complex, but with only 2 or 3 receptors per virion, it was not surprising that a 60-fold averaged whole particle tomogram at 10 Å resolution revealed nothing of the receptor.^83^ Automatic classification did not fare well, so bound sites in 1321 particles were marked manually. Subvolume tomograms yielded four classes with the expected particle size. The receptor appeared to be bound at a consistent site near the 3-fold spikes, radiating outward with increasing variation where there were not direct interactions with the virus. At ∼30 Å resolution, none of the protein detail is observable, so domains were modeled into the reconstruction as ellipsoids extending radially from trimers of spikes on the AAV2 surface. Additional distance constraints came from amino acid pairs that had been cross-linked (specifically for this experiment and not in the structure determinations) and then identified by mass spectrometry.^83^ Note that with a virus, there may be ambiguity between atomic models that can satisfy ∼15 Å cross-link distances because different symmetry equivalents of identified cross-linked amino acids can be brought into play. In retrospect (after the high-resolution structure was obtained), excessive allowance for structural flexibility had been made, and systematic searches for consistent atomic models could have been more stringent.^83^ The identification of several virus-receptor cross-links near the boundary between PKD1 and PKD2 provided a constraint, anchoring where an atomic model could be placed within a low-resolution tomographic map. With approximate models overlaid, it could now be seen that the four most populous classes from the tomography diverged after PKD3, with PKD4 and PKD5 turning tangentially but pointing in different directions. The hybrid application of x-MS and cryo-ET was providing useful structure for a highly flexible complex. Furthermore, consistency between the tomography, the subvolume cryo-EM SPA at 10 Å resolution, both using a five-domain construct, and later, the high-resolution structure of the two-domain construct, provided holistic evidence that the structural results were robust and not sensitive to technique or particular sample preparation.

#### 2.8.2 Details: Receptor Domains by Single Particle Analysis

The first detailed structures were complexes of AAV2 with AAVR. Even though one sample contained a PKD1–5 fragment and the other, a PKD1–2 fragment, both reconstructions revealed only the PKD2 domain that is bound most tightly.^82,83^ The PKD1–5 complex yielded a reconstruction with an FSC_0.143_ = 2.84 Å resolution.^82^ An atomic model was refined into this map using Phenix.^277^ The PKD12 complex yielded a reconstruction with an FSC_0.143_ = 2.39 Å resolution.^83^ This modestly higher resolution might have resulted from a complex with fewer flexible domains (perhaps easier to align particles) or a microscope at higher voltage (300 *vs* 200 kV), but data sets were of similar size (21 343 *vs* 16 820 particle images). Meyer *et al.* had attributed greater success with their PKD12 complex (*vs* their own efforts with MBP-PDK1–5) to more highly saturated binding achievable with a much smaller construct (occupancy refined to 0.48 *vs* 3% in PKD1–5 tomography), but now, seeing higher occupancy achieved by Zhang *et al.*, for their five-domain construct, it seems more likely that the MBP fusion domain affected the state of the complex.^82,83^

For model refinement of the AAV2–PKD12 complex, magnification was first calibrated by optimization against the AAV2 crystal structure.^83^ A preliminary model was built into the unsharpened map and refined using RSRef atomic refinement before rerefining into the sharpened map to yield a map-model correlation coefficient of 0.88 for map grid points within 2 Å of protein, water, and ion atoms.^83,261^ There is a modest systematic difference between the PKD12 and PKD1–5 coordinate sets that can be reconciled by refining a magnification for the PKD1–5 complex, and there is a trivial difference in residue numbering depending on whether the start is for native AAVR (Meyer *et al.*) or the expressed fragment which is 260 residues shorter (Zhang *et al.*). The structures are fundamentally very similar, and yet the papers come to quite different conclusions.

**Figure 11:**
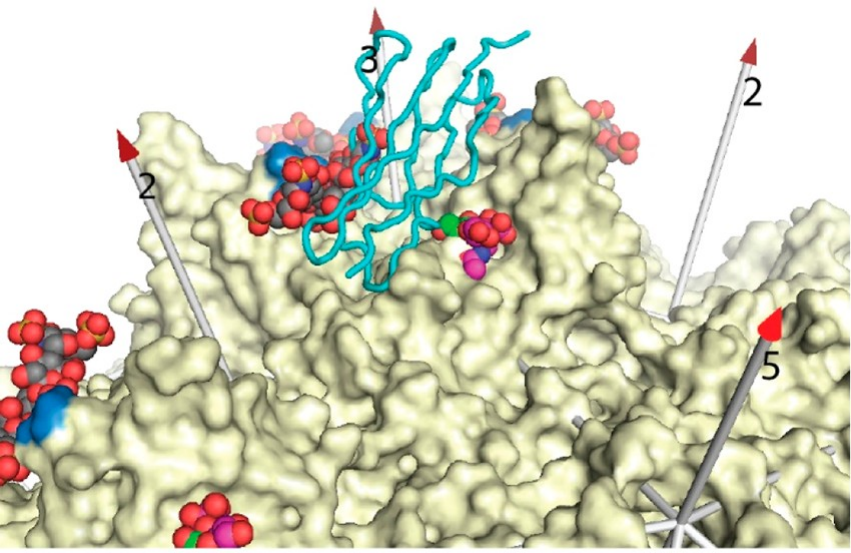
Conflict between the binding of the AAVR receptor and glycan attachment. Sites of glycan attachment are marked by overlaying on the surface of AAV2 the structures of fondaparinux, sialic acid, and galactose (in CPK sphere representation), bound respectively to AAV2, AAV1, and AAV9.^144,244,245^ Also overlaid is the PKD2 domain of AAVR from the AAV2 complex (cyan backbone trace).^83^ There is substantial direct conflict between fondaparinux (CPK grey carbons, left of AAVR) and AAVR near AAVR Asp_459_, indicating that heparan and AAVR cannot be bound simultaneously at the same symmetry-equivalent site on AAV. Galactose overlaps (CPK, right of AAVR) and sialic acid is close (binding at nearly the same site). Sialic acid and galactose are terminal residues on chains whose access would be obstructed by AAVR. All potential conflicts are with the PKD1 domain of AAVR. PKD2, as in the AAV5-complex, lies further away.

**Figure 12:**
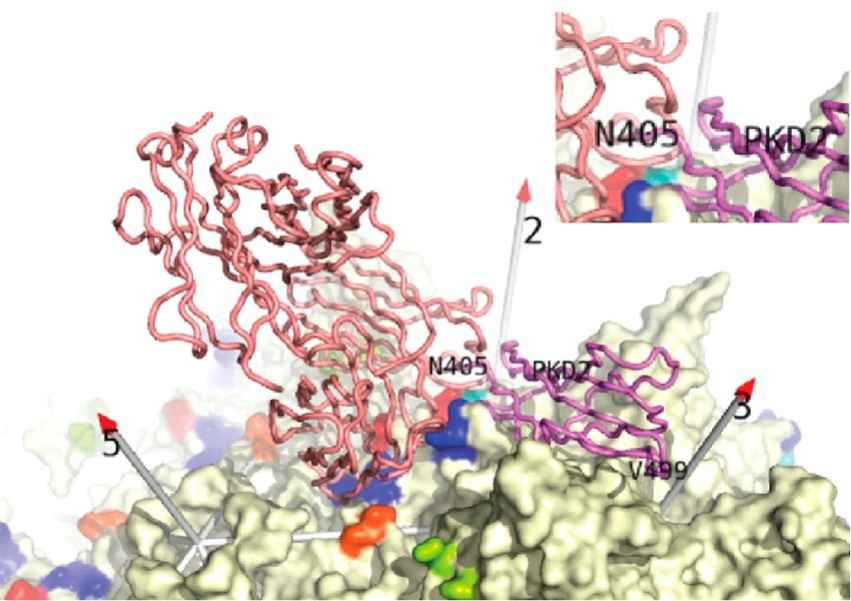
Conflict between the binding of neutralizing antibody A20 and the AAVR receptor. Overlaid on the surface of AAV2 are the AAVR PKD2 domain (violet) and the A20 Fab′ structure (salmon).^83,167,244^ The AAV2 surface is colored cream, except for A20 epitope amino acids (within 4 Å of Fab A20) that are colored by residue number from blue to red. The view is from the top of a 3-fold spike, down toward a 2-fold axis. Conflict (highlighted in the inset) is seen where both bind to the spur extending from the 3-fold proximal spike. The first AAVR residue seen is Asn_405_, but this implies likely conflict of mAb A20 with the unseen PKD1 domain: we see the first residues of PKD2 passing through space occupied by the CDR loops of the neutralizing antibody. Five residues of the AAV2 surface are part of the A20 epitope and also within the PKD2 footprint, but this view illustrates that conflict can also be several Å removed from the recognition surfaces.

Zhang *et al.* reported that the AAVR PKD2 binding site was far removed from both the HSPG binding site, and the mAb A20 neutralizing epitope, whereas Meyer *et al.* reported overlap with both.^82,83^ These are important distinctions affecting one’s understanding of whether glycan attachment and receptor-mediated entry compete and must therefore use different symmetry-equivalent regions of the capsid at each step (Figure 11). More importantly, it impacts one’s understanding of possible mechanisms of antibody neutralization. It is possible that the Zhang *et al.* analysis overlaid the structures in only single orientations because overlap in the Meyer *et al.* analysis became apparent when binding at sites related by the 60-fold viral symmetry were checked. So, even though the underlying structures are very similar, Zhang *et al.* conclude that their structure supports a proposed postentry A20 neutralization mechanism, whereas the analysis of Meyer *et al.* make a powerful case that the mechanism is likely to be competitive inhibition of receptor binding (Figure 12).^82,83,233^ It means that in modulating immune-escape in gene therapy vectors, care will be needed to avoid disrupting epitope-overlapping receptor-binding.

Central to AAVR binding are the surface loops, most prominently VR-I and VR-III on the plateau that are also the most prominent within the A20 epitope.^83,167^ Conformational changes induced by AAVR binding are local to the interface and were reported to be greatest within VR-I and VR-IV by Zhang *et al.* (see also below).^82^ Subsequently, the Tsinghua team published a 3.3 Å reconstruction of a complex with AAV1.^137^ AAV1 is quite close in sequence to AAV2, and the complex was very similar. Although domain deletion mutants had implicated PKD1 in addition to PKD2, the AAV1 complex again revealed only PKD2, more consistent with the viral overlay assay which indicated binding only to PKD1.^278^ Notably, within the βBC1-βC loop, also known as VR-I, the bound AAV1 conformation was like that found in the 2.4 Å AAV2 complex, suggesting that the changes in VR1 for both AAV1 and AAV2 were more modest than had previously been modeled into the 2.8 Å reconstruction. This is a reminder to all of us that an atomic model is an interpretation of a map which can be ambiguous in some locations. The significance of changes should be appraised not just through coordinate shifts but through differences in the underlying maps from which they are derived. This is perhaps even more important in cryo-EM than crystallography because of the wide range of local resolutions with which regions in the same structure can appear.

Both groups then proceeded to AAV5 because it was already known that the AAVR interactions were mediated through the PKD1 domain that had not been seen in the AAV2 complexes.^278^ First to be published was the 3.18 Å resolution structure from Tsinghua, followed by a 2.51 Å structure from Missouri, which was similar.^137,147^ The approaches followed those taken by the same groups for the AAV2 complexes, using the PKD1–5 and PKD12 AAVR constructs, respectively. The higher resolution structure was achieved using a 300 kV Titan Krios microscope (*vs* 200 kV Talos Arctica) and with 159 673 particle images (*vs* 12 590). A reconstruction of uncomplexed AAV5 was refined to 2.1 Å resolution, providing native structure with improved accuracy.^147^ Together with the 2.5 Å map of the complex, in which most side chains and carbonyls were apparent, a number of modeling ambiguities could be resolved, resulting in improved map-model consistency (CC = 0.88 for the 2.5 Å complex *vs* 0.81 at 3.18 Å). The rms difference between the Tsinghua and Missouri structures (1.3 Å) is larger than expected at 3 Å resolution. However, it would have been <1 Å had the magnifications of both reconstructions been calibrated against previously known structures. After factoring this out, rms differences range from 0.5 Å (AAV5 backbone) to 2.3 Å (AAVR all-atom).^147^ The latter are unexpectedly large and result from different atomic interpretations of weak map in the AAVR PKD1 domain near the viral 5-fold, with loops differing by 2–6 Å, and the N-terminus by up to 10 Å. The causes are discussed below.

**Figure 13:**
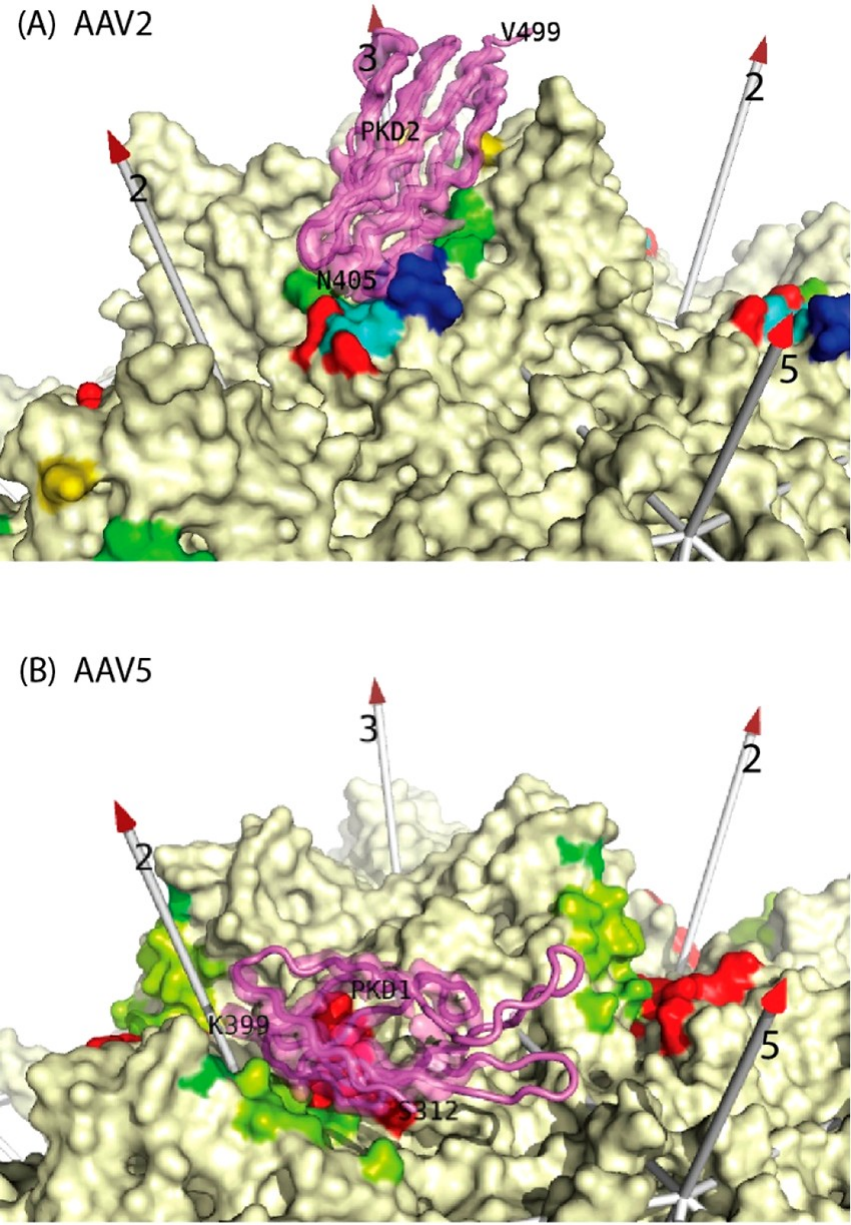
Structures of the AAV2 (A) and AAV5 (B) AAVR complexes taken from cryo-EM reconstructions at 2.4 and 2.5 Å resolution, respectively.^83,147^ The view is from above the canyon, between 2-fold (left) and 5-fold (right), looking toward the spur or plateau that leads up to a 3-fold proximal spike. The unsharpened cryo-EM reconstruction (translucent violet) is overlaid on the backbone trace, with a single domain of AAVR seen in each case: PKD1 with AAV2 (A) and PKD1 with AAV5 (B). Within the contact footprint (4.5 Å cutoff), the surfaces of AAV are rainbow-colored by residue number, distinguishing the variable regions (VRs). For PKD2, there is continuous map for the backbone and tight AAV interactions are concentrated near the N-terminus of the domain. For PKD2, AAV contacts are exclusively at the C-terminal end, with weaker map and greater disorder at the N-terminal end. The first residue expressed in this construct is Val_311_, but there are no AAV contacts near the 5-fold.

High resolution was not, however, needed for the most intriguing of results. Just as with AAV2, a single AAVR domain was seen, other domains present in the sample, but too disordered for visualization.^137^ The tightly bound domain was PKD1, in contrast to AAV2’s PKD2 but consistent with the earlier domain deletion mutants, viral overlay assay, and transduction inhibition assays in the presence of competing PKD1 or PKD2.^84^ Some had speculated that AAVs had a single PKD-binding interface that had adapted evolutionarily for binding to either of the homologous PKD1 or PKD2 domains. However, the PKD1 binding site was distinct, its N-terminal end above the virus 5-fold axis, its C-terminal end interacting with the side of a 3-fold spike facing of a 2-fold (Figure 13).^137^ Silveria *et al.* overlaid the AAV5 and AAV2 complexes, finding that the distance between the last residue seen in PKD1 of the AAV5 complex, and the first residue seen in the PKD2 of the AAV2 complex, on the other side of the 2-fold axis, was 19 Å and could not be bridged by any reasonable conformation of the linker residues that were not seen in any of the structures.^147^ Thus, the accessory role of PKD1 in AAV2 transduction could not be explained by imagining so-far unseen binding of PKD1 to AAV2 exactly as it was in AAV5.^246^ It is something of a mystery how AAV’s use of different receptor domains could have evolved.

**Figure 14:**
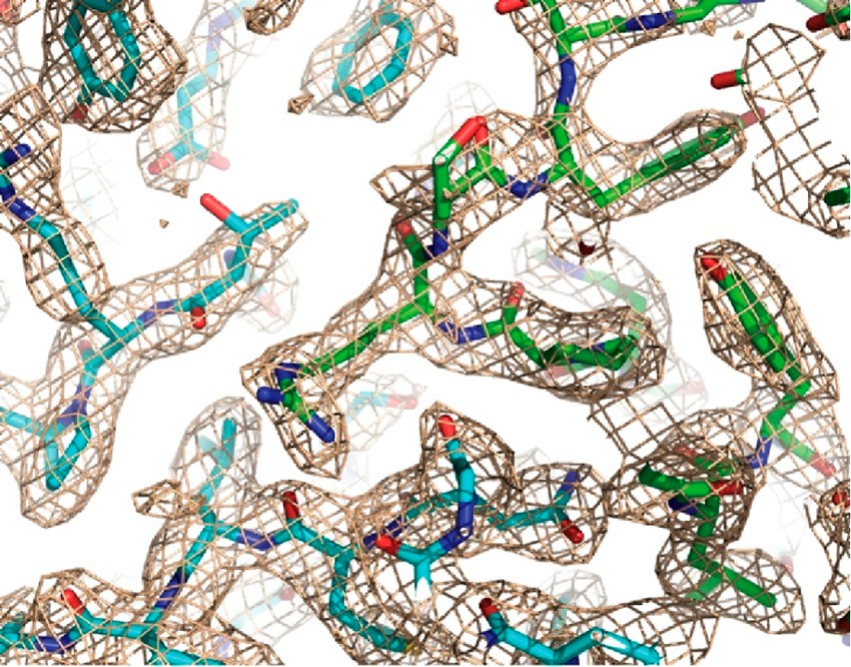
Cryo-EM reconstruction of the AAV5–AAVR complex. Notwithstanding disorder at the distal end of the domain (Figure 13), the map for AAVR PKD1 is well defined at 2.5 Å resolution. The view is centered on Arg_353_ of AAVR (green-carbon stick model), where it is surrounded by AAV5 (cyan-carbon stick model).

The PKD1 in both complexes is only partly ordered. The map at the C-terminal end of the domain, where it interacts intimately with AAV5 is very clear, with full side-chain detail (Figure 14). There is a gradual progressive weakening of the map such that the β strands fade out and loops are ambiguous or uninterpretable where the domain approaches the viral 5-fold axis. The higher resolution structure is no better. The appearance is consistent with the C-terminal end of the domain being anchored by interactions with AAV5, and then rotational disorder about this pivot point. Near the 5-fold axis, the map is too weak for independent modeling of the PKD1 backbone and should be guided by canonical homologues superimposed and anchored by clearer parts of the map where the C-terminal end of PKD1 is stabilized by its interactions with AAV5.

The Tsinghua and Missouri reports differ in their assessments of interactions between PKD1 and the 5-fold region of AAV5. The first describes an interaction between the N-terminal strand of PKD1 and the AAV5 loop that caps the 5-fold pore. The Missouri group finds no contact, but their construct starts at Val_311_, after the potential contact residues. In the Tsinghua structure, separate βA and βA′ segments have been modeled, linked by residues 313–5 of irregular structure. There is a clear map only for the C-terminal βA′. For residues 315 to 312, the Missouri group follows more conservatively the path of a homologue toward Val_311_ (the unseen N-terminal residue in their construct), through features that are stronger in their map. It is only in the Tsinghua model that βA is modeled and is interacting with the VR-II loop at the 5-fold axis, but their map in this region is very noisy.^137^ There is not strong experimental evidence for an interaction which should be considered tentative, conjecturally based on interpretation of a weak map features and without definitive mutational data. It could be speculated that the high disorder seen in both studies, at the N-terminal end of PKD1, suggests that there is not a stabilizing interaction.^137,147^

At the other end of the domain, there is mostly agreement in which regions are primarily responsible for binding. From PKD1, most of the interactions come from loops βC-βD and βD-βE and a lysine in βG. These make contacts with AAV5 residues primarily in VR-VII and parts of VR-IX.^137,147^ At amino acid level, 80% of each of the Tsinghua and Missouri AAVR footprints on AAV5 are in common with the other.^137,147^ A structure at 2.5 Å resolution supports plausible analysis of hydrogen bonding and other atomic interactions, and therefore some refinement of the footprint, but this affects only modestly analysis of potential conflict with other viral interactions.^147^ Apparent at high resolution, are the intimate contacts formed by Arg_353_ and His_351_ from the βC-βD loop of AAVR, residues that project into negatively charged pockets on the surface of AAV5.^147^ In both of their AAV2 and AAV5 reports, the Tsinghua team assayed phenotypes of single site mutations within the receptor footprints.^82,137^ This was particularly important for AAV5 because its footprint had not been probed extensively before.^242^ It is, of course, possible that the contribution of one amino acid might not have measurable impact upon transduction or viral overlay assay, but of 14 mutants tested, seven lowered transduction significantly, all but one common to the Tsinghua and Missouri footprints. Of those that had no impact, a disproportionate three of seven were outside the consensus part of the footprint.^137^

Changes in AAV5, induced by AAVR, are minor. In the interacting loops, nine amino acids differ by an rmsd of 1.0 Å, dominated by side chain movements of up to 3.4 Å.^147^ These are highly local to the binding site. The overall rsmd between bound and unbound is 0.4 Å (0.3 Å for backbone).^147^ Not only is this implying that the binding site is largely preformed, but it is indicating that the binding of AAVR is not sufficient to trigger longer range changes that might be needed for VP1u extrusion during endosomal trafficking.

As detailed earlier in this contribution, AAVR PKD1 lies over contact residues identified as within the epitopes of all monoclonal antibodies characterized to date: ADK5a, ADK5b, 3C5, and HL2476.^147,221,222,224^ These were separated by prior classification into neutralizing (ADK5b^222^ and HL2476^224^) and non-neutralizing (ADK5b^222^ and 3C5^249^). Silveria *et al.*, seeing the potential for conflict from the structures, assayed PKD1–ADK5b competition binding and found inhibition, albeit at higher PKD1 concentration than neutralizing ADK5a.^147^ One of the two contact sites for 3C5, the one thought recognized by the CDRs, is similarly situated, but was not followed up.^147^ In retrospect, the primary literature classifies Fab 3C5 as non-neutralizing and intact IgG 3C5 as weakly neutralizing.^249^ The latter inhibits cell transduction at 10- to 100-fold higher concentrations than AAV1 antibodies 4E4 and 5H7, comparable to the difference between ADK5a and ADK5b.^222^ On reflection, perhaps we need to be careful of qualitative distinctions, with a message from the structures that all of these antibodies have the potential to interfere with receptor binding. Further evidence is needed to understand which could be neutralizing at physiologically relevant concentrations and whether such interference is a dominant mechanism. Integrating the results from AAV1, AAV2, and AAV5, neutralization mechanisms have been thought to be postentry for a number of antibodies.^210,221,222^ The structural studies of receptor complexes, along with better understanding of the distinction between cell-attachment receptor-mediated entry, forces reevaluation of the evidence behind the dogma. Not to the exclusion of other possible mechanisms, we now see that interference with receptor-binding is plausible for many of these antibodies.^147,279^

## 3 AAV: A Driver and Beneficiary of EM Advances

High interest in AAV has motivated application of the latest EM technologies to the cause, with AAV often serving as an early test case, and even, at some junctures, driving the technology development. Early application of emerging technology to AAV has highlighted both opportunities and limitations, with lessons for improved practices that are more broadly relevant in structural biology. This section highlights the technological underpinnings behind the advances in structural virology discussed in the previous section and the potential for AAV to continue serving as a test case in EM development.

### 3.1 Tractability: Cryo-EM Replaces Crystallography in AAV Structural Biology

In their recent completion of an atlas of AAV structures, the group of the late Mavis Agbandje-McKenna added structures of four serotypes, representing clade D and other lineages.^86^ All structures were in the 2.5–3.0 Å resolution regime that is accessible to crystallography, but all were attained by cryo-EM. The cryo-EM results are often equal, if not better, and an EM structure is usually much easier and quicker to obtain.

The very first AAV atomic structure (by X-ray crystallography) took about 20 person-years of effort, and now an AAV EM structure is sometimes only one person-year of effort. Efficiencies were realized in later crystallographic studies, but AAV structural virology accelerated markedly when crystallographic-like resolutions became achievable by EM. The first challenge in AAV crystallography had been virological scaling up of microgram preparative methods to the milligrams needed for crystallization screens.^78^ Much smaller quantities are needed for cryo-EM that are more readily available (as detailed above). Furthermore, virus crystallography is never routine, but the asymmetric repeating unit of the AAV2 crystals contained three complete virions, making it one of the largest and technologically challenging structures eversolved.^280^ Subsequent serotypes could be boot-strapped from AAV2, but sequence differences led to different crystallization conditions and packing configurations, so structure solution was never particularly straightforward.^80,148,281−283^

Given such challenges, there was high interest in early adoption of alternative approaches. Technological advances in cryo-EM over the past decade have been remarkable, still continue, and are reviewed elsewhere in this volume. By about five years ago, they combined to make possible structure determinations of AAV at 3 Å resolution or beyond. Key improvements included direct-electron detectors to significantly boost the quality of individual images, collection of movies for correction of specimen drift and beam-induced motion, more optically stable microscopes and automation for long, high-throughput data collections, and vastly improved algorithms for alignment, classification, and correction of imaging aberrations to higher resolutions.^256,284,285^ With already extensive experience in preparing suitable sample (above), AAV was primed to take early advantage of technological improvements, and the foundations have been in place for AAV to become a popular and approachable model system.

**Figure 15:**
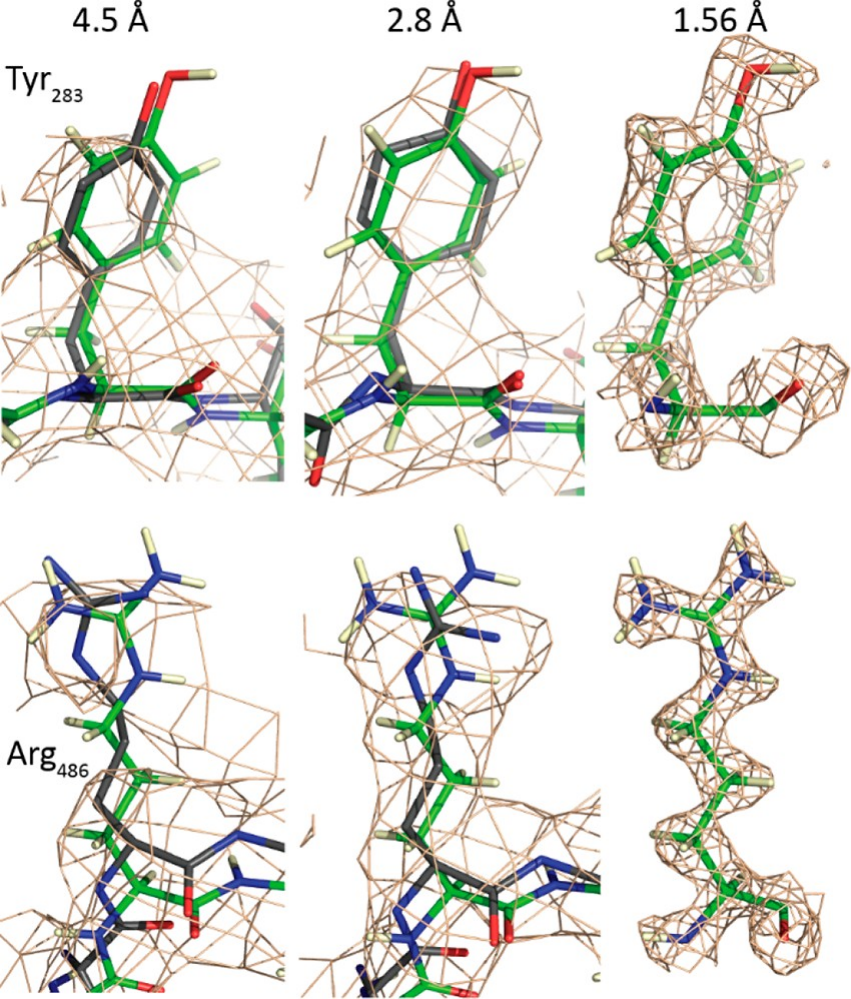
AAV-DJ Coulombic potential maps at different resolutions. Tyr_283_ is buried and is an example of one of the more ordered side chains. Arg_486_ is a surface residue that are typically less well ordered. Structures of AAV-DJ have been determined at different resolutions, at 4.5 Å, at 2.8 Å (in complex with fondaparinux), and at 1.56 Å.^128,132,244^ The structure refined at 1.56 Å resolution is shown with green carbons, with structures refined into lower resolution maps shown with gray carbons. Backbone map is continuous at all resolutions through most of the structure, allowing a complete trace. At 1.56 Å resolution, carbonyls, hydroxyls, and many hydrogens are apparent, defining unambiguously peptide dihedrals and hydrogen bonds. For the well-ordered Tyr_283_, the map is sufficient to model a constrained aromatic side chain well at 2.8 Å, and enough is apparent even at 4.5 Å resolution. Although the fit of Arg_486_ appears good at 2.8 Å resolution, the map is truncated and the functional guanidinium atoms are misplaced by >1 Å, so designation of salt bridges would not be robust. While at 1.56 Å resolution, the extended conformation of Arg_486_ is clear, the more bulbous map at 2.8 Å allowed a shorter “corkscrew” rotamer to pass muster compatible with a somewhat misplaced backbone. At 4.5 Å resolution, the map is broken and connectivity wrong near Arg_486_ C_δ_. The model is a reasonable approximation only because it was built using a high-resolution crystal structure and refined using a conservative algorithm,^13,261^ otherwise, side chains at ca. 4 Å resolution are often plausible guesses among commonly occurring rotamers.

**Figure 16:**
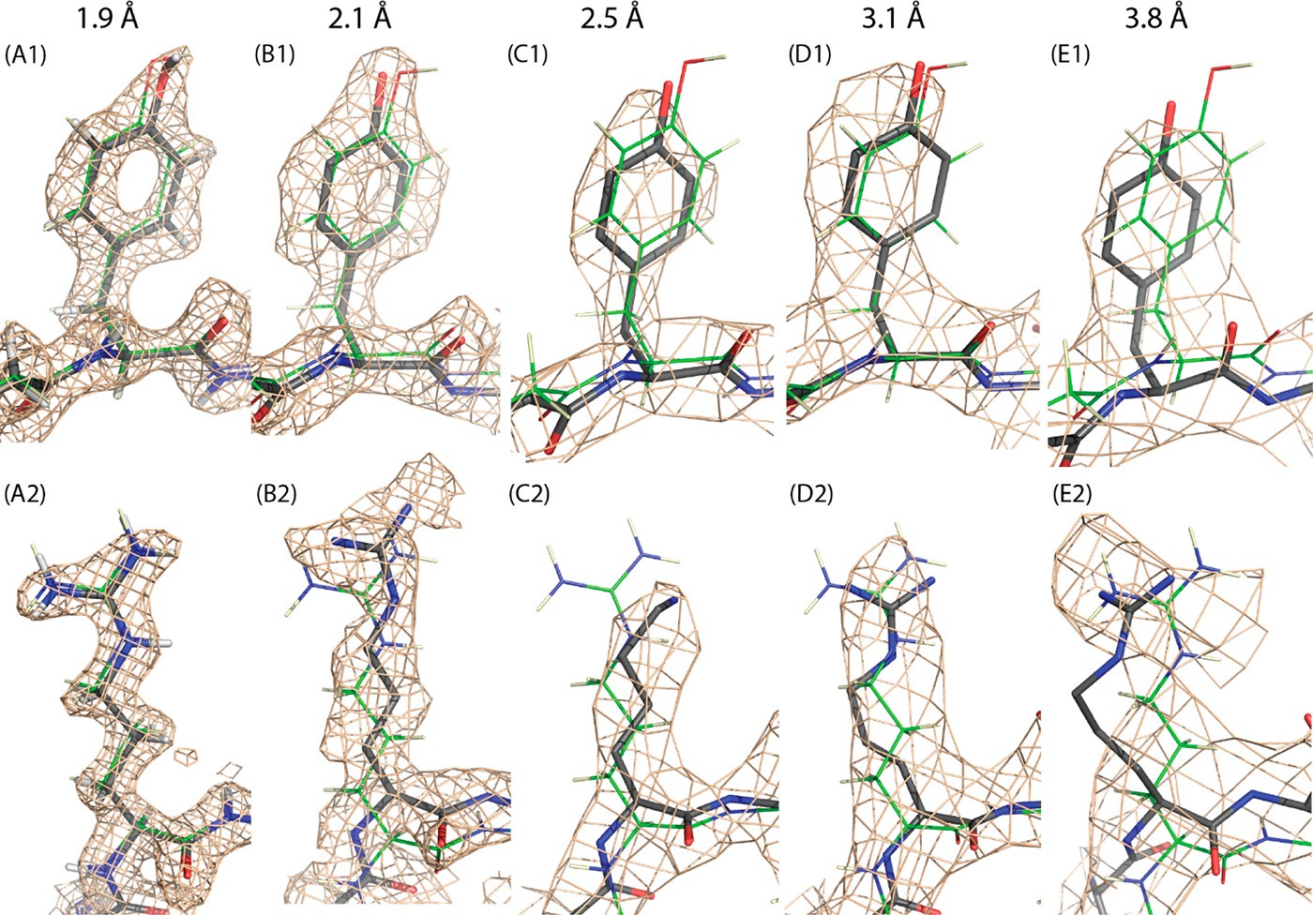
Coulombic potential maps from the structures of different serotypes exemplifying representative resolutions. This figure illustrates ambiguities and errors that would be typical for AAV structures at the stated resolutions. Illustrated amino acids align with those of Figure 15: an interior tyrosine, expected to be well-ordered and among the clearest (1) and a surface arginine or lysine, expected to be less well ordered (2). Atomic structures are shown with gray carbons, with the structure of AAV-DJ^128^ superimposed (1.56 Å, green carbons, PDB 7kfr). (A) AAV2 L336C is an excellent fit at 1.86 Å resolution (PDB 6e9d, EMD 9012^129^). This structure was determined completely independently of AAV-DJ. Close agreement indicates that AAV-DJ can be regarded as a ground truth comparator for the non-hydrogen structure of other serotypes in regions (Tyr_284_ and Arg_484_), where sequence differences have no impact. Comparing this 1.86 Å map to the 1.56 Å (Figure 15), we see some, but fewer of the hydrogens. (B) At 2.1 Å resolution, AAV5 Tyr_272_ shows no evidence of hydrogens but is otherwise modeled well (PDB 7kp3, EMD 22987.^147^) Arg_484_ is slightly less well defined but clearly different from AAV-DJ. Map ambiguity forces a choice between a model centered at the backbone or moved 1/2 Å for seemingly better fit of the guanidinium group. (C) At 2.5 Å resolution, there is no doubt of the identity of Tyr_281_ and Lys_487_ in AAV12 (PDB 7l6b, EMD 23201^86^), but the tyrosine is missing some density; the backbone of Lys_487_ lacked the definitive features to see, in retrospect, that a structure more like AAV-DJ would have been ∼0.3 Å better. (D) In the 3.1 Å structure of AAV1 (PDB 6jcr, EMD 9795^137^), local constraints yielded a correct model for Tyr_282_ in spite of missing map for C_δ_ and C_ε_. Truncated map for Arg_485_ led to a “corkscrew” rotamer model that we would not choose in retrospect. (E) The 3.8 Å wtAAV2 structure (PDB 5ipi, EMD 8099^133^) is expected to be near identical to the L336C mutant (A). Fitting the side chain of Tyr_281_ into a truncated map has led to a ∼1 Å deformation of the backbone. At slightly higher contour, the map for Arg_484_ is discontinuous, with the result that the rotamer is incorrect, and compensating deformations of ∼1.5 Å have been made in the backbone. Inverting the narrative, even below 4 Å resolution, the approximate backbone path is clear. The general directions of side chains become clear and backbone more precise between 4 and 3 Å resolution. Rotamers become unambiguous between 3 and 2 Å resolution. Beyond 2 Å resolution, most non-hydrogens will be accurately placed, and with further improvement in resolution, more of the hydrogens become clearly defined.

The recent structures between 2.5 and 3.0 Å have illustrated how tractable AAV structures by cryo-EM have become. Several of the ∼3 Å structures above were determined using less than 1000 images and 10–30 000 particle images using Titan Krios 300 kV microscopes with CMOS direct electron detector devices (DDD) that are now installed at a number of institutions and accessible at National Centers supported by the National Institutes of Health. Cryo-EM structures at these resolutions are very much what you would expect at corresponding crystallographic resolutions. In well-ordered regions, differences between (for example) aromatic and aliphatic side chains are clear enough for reliable fitting of the chemical sequence, and while the backbone configuration can normally be inferred reliably, at worse than 2.5 Å resolution, there is not yet direct observation of carbonyl orientation for most peptide bonds (Figures 15 and 16).

### 3.2 Atomic Resolution Structures

It is often said that it is symmetric and well-ordered macromolecular assemblies, like apoferritin, that are most amenable to high-resolution cryo-EM.^286,287^ For AAV, the symmetry is high (60-fold) for much of the assembly, but parts do not conform or are disordered: VP1, VP2, and internal nucleic acid. Thus, it runs somewhat against conventional wisdom that AAV is the biomolecular assembly currently running second only to apoferritin in terms of the cryo-EM resolution achieved.^128^ The reasons that our unlikely candidate has become particularly amenable to the highest resolution studies will be discussed elsewhere. Here, we consider common denominators between efforts beyond 2 Å resolution, and what they imply for tractability, and we consider the structural virology learned from these particularly high-resolution structures.

The two independent studies used baculovirus-expressed empty capsids of either the L336C mutant of AAV2 or the AAV-DJ vector.^128,129^ Both used high-end Titan Krios microscopes, without phase plates and with now dated configurations that would no longer be considered state-of-art. Image-shifting and fringe-free data collection were not available. The studies differed in use of: (a) gold or copper sample grids, (b) second or third generation direct electron detectors (Gatan K2 Summit or Thermo Fisher Falcon 3), (c) objective Cs spherical aberration correction, and (d) pixel sizes of 0.51 or 0.79 Å (0.39 Å in super-resolution mode). One cannot rule out the importance of particular combinations of these factors, but the lowest common denominator suggests that atomic resolution should be achievable on microscopes that are now accessible to many. In both cases, application, toward the end of processing, of an Ewald sphere curvature correction for intraparticle focus gradient produced recognizable improvement.^288,289^

At 1.86 Å resolution, Lyumkis and colleagues reported holes in aromatic rings, a crystallographer’s indication of quality. More importantly, they reported side chain definition explicitly indicating rotamers, carbonyl oxygens (and proline puckers) explicitly defining backbone conformation, waters of solvation shells, and the first indications of hydrogen atoms.^129^ By 1.56 Å resolution, the Chapman group report now that most of the hydrogens and protons can be observed.^128^ They quantify that, on average, the signal for solvent waters is about half of that for ordered protein, and that, by this resolution, hydrogens are emerging at about a quarter of the strength that would be expected of unlimited resolution. This is sufficient to resolve ambiguities in the rotamers of pseudosymmetrical side chains like histidine, asparagine, and glutamine because amine groups are distinguished from carbons and oxygens. Further gains will be possible, but even 1.56 Å provides enough clarity for direct observation of much of the hydrogen bonding network. Within the better parts of the structure (including just over half of the histidines), the protonation state of titratable atoms is discernible, providing direct evidence for salt bridge interactions, charge states, and where lone pairs are free for ion coordination. Crystallographers may be surprised that hydrogens can be observed even at 1.6 Å resolution. This is due to the greater scattering of electrons by nuclear charge than of X-rays by the single electron of hydrogen atoms.^286,290,291^ The details revealed at higher resolution are potentially important in understanding viral assembly, stability, (environmentally dependent) conformational transitions, and molecular interactions with host factors. The relevant complexes have not yet been subject to such high resolution studies, but it is sobering to note the examples cited by Xie *et al.*, of rotamers and hydrogen-bonding within AAV-DJ, inferred at 2.8 Å resolution, that, in retrospect, we now see as unreliable.^128^ It is not widely appreciated that resolutions between 2.5 and 3 Å allow models to be built with plausible hydrogen bonds but do not yield models of sufficient accuracy to ascertain reliably whether there is actually an attractive interaction. Thus, there will be a continuing need to pursue selected AAV structures at sufficiently high resolution for robust atomic-level characterization of molecular interactions.

### 3.3 Complexes

Of central interest is how AAV capsids interact with host molecules. A number of technical challenges first became apparent in studies of antibody binding but are also relevant to complexes with receptors. Problems are exacerbated at low resolution, which is often the reality with complexes: 12 of the 14 AAV-antibody complex structures are at resolutions worse than 6 Å (Table 2). It is not just a question of legacy structures that predate the “resolution revolution” but the same flexibility and conformational heterogeneity that precludes crystallization of such complexes often limits cryo-EM resolution.

#### 3.3.1 Intermediate Resolution and Pseudoatomic Models

Many of the challenges first became apparent with the AAV2:A20 complex, which has shaped the approach to other complexes at even lower resolution with potentially greater pitfalls.^167,234^

##### 3.3.1.1 Difference Maps

In the 3D cryo-EM of complexes, a ligand is often weaker than the target. Binding might be less than saturated or there might be disorder/flexibility in the ligand. The map of the ligand is then weak because all sites are assumed to be identical in the application of icosahedral symmetry during reconstruction. A more sensitive visualization is provided by difference maps, calculated by subtracting, after scaling, a native state reconstruction from that of the complex. Early attempts with AAV2:A20 were confounded by two issues. First, a 7.5% miscalibration in magnification had led to artifactual differences between mismatched reconstructions of native and complex. These artifacts dominated difference maps until the error was discovered and corrected.^167^ This was an unusually large error, but a magnification error of 4% was reported by Gurda *et al.*, and errors of 2% remain common, resulting in artifactual features if not corrected.^221,261,292^ Errors in magnification (or pixel size) most commonly result from experimental errors in calibration (using gold diffraction). Pixel size estimates are more accurate following refinement of a reconstruction against a known (crystal) structure.^261^ Difference maps are affected when the systematic errors in complex and native are different, most likely when the data sets have been collected with different microscopes or configurations.

Second, the “density” values (more properly Coulombic potential values) in any particular map are on an arbitrary scale depending on many experimental and processing parameters. A difference map will show exclusively ligand, only if the “densities” in the target are matched. Most commonly, the gain of the two maps is calibrated by matching the mean and standard deviations of the entire map volume. There are several reasons why differences in the mean and variances should be expected, most obviously when the ligand occupies significant volume (that would be solvent in the native reconstruction). With the AAV2:A20 complex, appropriate difference maps were obtained only when the scaling coefficients were calculated exclusively from the region of the capsid protein common to both reconstructions, then applied to the entire volume.^167^ Such masked scaling is performed in some studies but not all. Even better, when a high-resolution structure of the target is known, both maps can be scaled to a common reference atomic model. One of the approaches for doing this allows for the reconstructions to differ in effective resolution (due to disorder or different sized data sets), mitigating the worst of resulting artifacts.^258,261^

Difference maps are prone to misinterpretation if the features are weak and/or do not have shape specifically characteristic of the molecule. By definition, there will always be differences in the pixel values between two experimental maps, whether they be real, experimental noise, or systematic artifacts. At the 8 and 18 Å resolutions of the competing studies of heparin complexes,^243,259^ maps feature no details of chemical structure that, at high resolution, provide internal validation of molecular interpretation. These are resolution regimes where great care is needed. Furthermore, in the 18 Å study, features were weak (0.5 to 1 σ) and difficult to distinguish signal from noise.

##### 3.3.1.2 Homology Modeling

While there is an alternative approach (*vide infra*), most structure-based epitope mappings rely on fitting an Fab atomic model. High-resolution component structures are usually known for the virus, but not usually the antibody. At resolutions below those needed to build one into the EM reconstruction, *de novo*, the model to be docked must be predicted by homology to related proteins of previously known structure.

The standard for structure prediction has been software like Modeler with multiple templates, perhaps applying it locally using homology of individual CDR loops to others of similar length.^293^ Best practices have recently been consolidated in the WAM algorithm: backbone conformation is guided by prior structures for canonical class CDRs, and a combination of knowledge-based and *ab initio* prediction is used for the backbone of noncanonical loops (*e.g.*, CDR3), before searches for energy-optimal side chain rotamers.^250^ Tests with known structures show canonical loops with rmsd errors of 0.4–3.7 Å and typically 2 Å for noncanonical loops.^250^ Structure prediction methods are improving rapidly with deep learning and neural network algorithms.^294−296^ However, these latest generation methods use coevolution as a predictor of close proximity, exploiting (hidden) constraints on sequence diversity in ancient families of homologous proteins. The authors are not aware of evidence that a similar approach is applicable to the all-important Fab CDRs that evolve by somatic hypermutation and affinity selection.^297,298^ Improvement does not appear on the near horizon, and we must consider the combined errors of homology modeling and map-fitting. The latter dominate at nanometer resolutions where cryo-EM shows just a molecular envelope. At intermediate resolutions, accuracy is limited by homology modeling until fully resolved backbone and side chains remove the need for prediction.

##### 3.3.1.3 Fitting Component (Antibody) Domains into an EM Reconstruction

Here we are concerned with how we are guided by the map in the optimal docking of known component (homologue) structures, using as an example the docking an antibody Fab domain with the virus. Computer programs, such as Chimera or Situs, have routines for optimizing the fit of an atomic model within a reconstruction, but these should not be the first line of attack in optimizing the (rigid-body) fit of a virus subunit to a low-resolution reconstruction.^299−301^ It is more likely that any starting mismatch is due to an uncorrected miscalibration of magnification than there is a real perturbation of the viral quaternary structure induced by ligand-binding. Without any degrees of freedom, a prior atomic model of the native AAV can be aligned by overlay of the viral symmetry elements, and then the magnification refined before determining what further structure refinement might be indicated.^261^

Even at very modest resolution, good approximations to interaction footprints on the viral surface (such as epitopes) can be achieved because the underlying virus structure has previously been determined at high resolution, and, to a first approximation, can be assumed to be unchanged in a complex. More generally, and even absent prior high-resolution component structures, constraints come from atom connectivity in a known protein sequence and geometric expectations from principles of stereochemistry. In other words, we can improve the accuracy of atomic models beyond the experimental EM resolution by also incorporating *a priori* information.^302^ If both fit to the map and stereochemistry are optimized together, using a model parametrization appropriate for the resolution, test cases show model accuracy can surpass map resolution by 3- to 5-fold (depending on refinement approach).^128,261^ Well-executed, a 15 Å refinement can be accurate to ∼3 Å, the approximate separation of amino acids along a peptide chain. At 6.7 Å (like the AAV2:A20 complex) atom position errors should be ±∼2Å, sufficient to identify correctly most interacting amino acids but insufficient for atomic level interpretation. At these resolutions, rigid domain refinement is indicated because individual chains are not resolved and there is no basis for flexible fitting.

With an Fab optimally docked to the virus using the reconstruction, there is still the question of which virus amino acids form the epitope contact surface. As one lacks the resolution to model otherwise, it is usually assumed that conformation of amino acids at the interface are not greatly affected by the interaction. Then one lists AAV amino acids whose atoms come closer to the Fab than a distance cutoff (say 4 Å).^167^ This is subjectively larger than 2.5–3.0 Å theoretical interaction distances, to give some allowance for the substantial experimental errors.

##### 3.3.1.4 Model-free Epitope Footprinting

An alternative approach uses an atomic model for the virus, aligned by symmetry, as above, but then designates the epitope directly from the EM reconstruction without reference to an Fab atomic model.^261^ A threshold in map value of the reconstruction must be decreed as indicating contact. This might be the contour level that encloses the volume expected of the ligand, assuming that disorder and map strength are uniform.^167^ The limitation of map-based foot-printing is that features or gaps between molecules are not discerned beyond the nominal experimental resolution. Rarely will model-independent foot-printing be more accurate, even compared with the combined errors of homology-modeling, map-fitting, and distance thresholding, but the approach can offer a somewhat independent cross-check on model-based analysis.

Both approaches were applied to the AAV2:A20 complex, and the footprints agreed on a core epitope of 12 amino acids, with an additional five on the periphery suggested by map coverage, or four by contact (<4 Å) with a homology model.^167^ Thus, there was 60% agreement between the methods. Homology modeling supplemented with a database of CDR structure changed 20% of the contacts, but the agreement with a model-free footprint was improved by only 5%.^167,303^ The lessons are that: (i) the core of an epitope can be identified robustly at intermediate (7 Å) resolution, (ii) the exact boundary of the footprint is a method-sensitive approximation, and (iii) *pseudo*-atomic models are insufficient for hydrogen-bonding and other atomic analyses.

### 3.4 Comparative Analyses

AAV is typical of many studies in that interest extends beyond initial structure to subtleties that might explain phenotypic differences between variants or changes resulting from molecular interactions. There are two approaches. Each reconstruction can be interpreted with an atomic model and atomic coordinates analyzed. Alternatively, maps can be compared directly to highlight the greatest differences. AAV has shown that there are advantages and limitations of both approaches, and experience with AAV has led to development of better practices.

#### 3.4.1 Comparison of Atomic Models

It is very common to infer significance from the magnitude of coordinate differences, usually expressed as a root-mean-square deviation (rmsd). There are multiple potential problems. First, there is rarely consideration of the underlying experimental uncertainty of the coordinates. In large part, this is because there are no widely accepted methods for estimating the coordinate errors for EM structures. Test-case assessments against independently determined high resolution yardsticks indicate that appropriately restrained high quality refinements can have average coordinate errors that are 5-fold lower than the nominal resolution (all atom) with backbone errors about half of that.^128,261^ Thus typical average errors at 3 Å are ∼0.75 Å, and the error on a distance measurement (providing that the coordinates are independently determined) would be ∼0.75 × √2 = 1.1 Å. Anything less than this (or 0.6 Å for backbone) should be considered insignificant at 3 Å resolution. There is much local variation in coordinate accuracy with points of interest often on the less ordered molecular surface where the thresholds of experimental significance should be higher.

Systematic errors also affect calculation of RMSDs. An uncorrected 2% miscalibration of EM magnification results in a ∼2.5 Å systematic error in coordinate positions. This is largely factored out if RMSD is calculated after least-squares superposition of subunit coordinates, as is usually the case. Also, the numbering of carboxylate oxygens is always arbitrary, but at resolutions worse than 1.8 Å, carbons, oxygens, and amino groups are indistinguishable, so side chain flips (χ rotations of 180° at sp^2^ hybridized atoms) should be checked for closer agreement lest rmsd be artificially inflated. Most programs do not check this automatically.

The natural desire for structures to provide functional insights continues to incent interpretation of differences that have marginal statistical significance. It usually only comes to light in rare cases where structure determinations are repeated independently. Examples would include the extent of structural changes in AAV2 induced by the binding of heparin or AAVR.^82,83,243,244,259^ Until structural biology practices improve, it is unfortunately the consumer’s responsibility to question whether a coordinate difference is likely significant at the experimental (local) resolution. It is possible that there are other structure-based conclusions in the literature that need to be subject to such review. Difference maps (next section) have their own problems but offer a model-independent appraisal of conformational change. Confidence will be greatest when coordinate and difference map analyses offer a consistent interpretation.

#### 3.4.2 Difference Maps

Difference maps are of two types and can be used in three contexts. Maps that are the difference between an experimental reconstruction and a fitted atomic model are used to highlight needed improvements in an atomic model. Methods for the latter are under active development and are not considered further here.^304^ Maps, calculated as the difference between two experimental reconstructions, can be used to discover the binding mode of a ligand as described in section 3.3.1.1 or, as described here, to highlight conformational change in a model-independent way.

Difference maps can be very sensitive but amplify both systematic and random errors in ways that the community is learning only gradually. For difference maps, it is critical that: (1) magnifications be calibrated if the data sets have been collected with different microscopes/configurations, (2) relative intensities (baseline and gain) of the two maps are properly normalized, because the electrons/Å^3^ are on arbitrary scales, and (3) resolutions (and attenuation with frequency) be similar so that there are not systematic differences in Fourier series termination artifacts. All of these, but particularly the last, are important on minimizing the risk that artifacts or noise are mistaken for biologically interesting differences.

Gerlach *et al.* made extensive use of difference maps in their low resolution analysis of the rAAV1 capsid dependence on DNA packaging state.^85^ Several potential problems were mitigated by the authors’ care in comparing reconstructions calculated from different particle subsets within the same image data set. Thus, there should not be systematic differences in magnification, defocus, quantum detector efficiency, or other instrumental parameters. Nevertheless, the authors concluded that there were DNA-dependent changes in the capsid that have not been seen in subsequent higher resolution studies, illustrating the challenges in such analyses.

More often, difference maps are calculated between reconstructions from different samples. Then, it is particularly important that the maps be scaled in magnification and amplitude as described in section 3.3.1.1. A conformational change should lead to characteristic side-by-side positive and negative features, negative in the region whence the structure started, positive whither it moved. A discrepancy in magnification can have a similar effect, and the two can be difficult to distinguish in the presence of experimental noise. Poor scaling of amplitude can also lead to positive or negative artifacts that are easy to misinterpret. Differing attenuation with resolution can lead to Fourier series termination ripples in a difference map. This has led us to attempt difference map analysis only with data sets collected with similar microscope and detector configurations. Joseph *et al.* have introduced methods for scaling reconstructions by Fourier shell prior to difference map calculation.^304^ It is not yet clear to us what range of artifacts one should expect to be mitigated by such computational corrections. Finally, it is noted that difficulties are compounded at resolutions below those needed to see characteristic features of expected chemical groups because it is much more difficult to verify that features in the difference map are real and not artifactual. At low resolution, one seeks outside corroborating evidence, generally of the biological implications.

### 3.5 Heterogeneous and Flexible Elements by Cryogenic Electron Tomography (Cryo-ET)

Cryo-ET is a variant of cryo-EM that under very active development has recently been applied to structural studies of AAV to characterize the substantial heterogeneity in AAV/AAVR complexes. Cryo-ET applies low dose imaging to a rotating sample, providing 3D information for every particle, with advantages in classifying and aligning when there is significant heterogeneity.^275,276^ The tradeoff is the lower signal for each image, and generally lower resolution of final tomograms compared to cryo-EM single particle analysis (SPA). Note that, heretofore, AAV cryo-ET has mostly been applied to isolated (purified) assemblies, just like cryo-EM SPA, and not to the *in situ* visualization of cell contents, another application enjoying rapid progress.^305^

The sample is rotated over a range relative angles, typically from −65° to 65°, in known increments, but due to imperfections during data collection, there is typically some additional shift from image to image, so the images are then aligned relative to each other and back-projected to yield a 3D volume of the sample of interest. This technique is particularly useful for deriving (relatively) high-resolution 3D information for samples that are comprised of a heterogeneous field of biological assemblies and even samples like thin sections of cells.^305−307^ However, the technique has many downsides that limit the ultimate resolution of tomographic reconstructions. First is the dose. Biological molecules are sensitive to the radiation resulting from exposure to the electron beam in the TEM. An accumulated dose of 10 e^−^/Å^2^ can limit the high-resolution features of individual cryo-TEM images to ∼8 Å.^308^ Generally, the largest dose a sample can tolerate before losing all semblance to its original structure is around 100 e^–^/Å^2^. However, cryo-ET requires repeated imaging and dosing of the sample. In practice, during tilt-series data collection, the 100 e^−^/Å^2^ dose is fractionated over the full tilt series such that no image receives more than ∼2 e^−^/Å^2^. The consequence is that individual images of the tilt series are extremely noisy. This makes it challenging to align them relative to each other, further limiting resolution of the resulting 3D reconstruction. The second major factor that limits tomographic resolution is the focal gradient resulting from tilted samples. Contrast is generated in cryo-TEM by slightly underfocusing the microscope during imaging, enhancing the phase contrast. However, the defocus results in ripples in the (Fourier space) contrast transfer function (CTF) that produce frequency dependent inversions in the contrast of the image unless perfectly corrected during computational data processing. The CTF can be well described for images with 0° tilt and can be nearly completely corrected. However, for tilted images with a focal gradient, the CTF correction is much more challenging (1) because the magnitude and direction of the focal gradient must be determined and locally corrected, and (2) because the dose is so low for individual tilt-series images that there is very little signal with which to estimate the CTF. A final major limitation is that because the thickness of the sample increases with tilt, there is a limit to how steeply the sample can be tilted, usually no greater than ±65°. This results in a “missing wedge” of data from the 3D reconstruction.^309^ The resulting 130° data set can be thought of as intermediate between a complete 3D visualization and a 2D image that corresponds to a projection of the sample. The missing wedge thus produces artifactual distortions in the 3D reconstruction with features elongated along the microscope axis and/or missing altogether. Together, these factors combine to limit how much high-resolution information can be derived from tomographic maps.

Many of the limitations of cryo-ET can be overcome by merging data (in masked Fourier space) from different subvolumes of the tomogram containing the same object, thereby mitigating missing wedge effects and improving signal-to-noise ratios in so-called subtomogram averages. Assuming the CTF is estimated correctly and enough copies of the specimen of interest can be identified, subvolume averaging can be used to produce near-atomic resolution reconstructions of the specimen of interest from tomographic tilt-series, although this is currently the exception for favorable cases rather than the rule. A seminal example of this approach was given in Schur *et al.*, where the authors used tomography and subvolume averaging to determine the structure of the immature HIV-1 CA-SP1 lattice at 3.9 Å resolution.^310^ In that case, the authors benefitted from the many copies of the CA-SP1 in the lattice to improve the averaging and resolution of the subvolumes. However, the work shows that if enough copies of a specimen of interest can be identified and averaged, the tomographic approach is capable of near-atomic resolution.

Another advantage of subvolume averaging is that it can enable classification of heterogeneous specimens that are too varied for single particle analysis. Because whole volumes are analyzed instead of projected 2D images of the specimen, it is possible to uniquely isolate the heterogeneous regions in three-dimensions and structurally characterize the different conformations and compositions. This approach enabled characterization of the substantial heterogeneity present in complexes of AAV with the full-length AAVR receptor ectodomain. The linkages between the PKD domains comprising the AAVR protein are largely unstructured, giving the protein many degrees of freedom. Nonetheless, it was found that AAV/AAVR complexes assume a handful of stable conformations.

### 3.6 AAV as a Methods-Development Model System

AAV has qualities that make it an ideal model specimen for methods development for cryo-EM. First off, it is rugged. It freezes well, and very few particles appear to be damaged during blotting and vitrification. Second, images of AAV are featureful. Compared to the other leading model specimen in the cryo-EM field, apoferritin, it has clearly identifiable features at both the high and low-resolution regimes. For instance, before the introduction of direct electron detectors, apoferritin could not be confidently reconstructed while high-quality single particle reconstructions of AAV have been possible since the cryo-EM field used CCD detectors. Another advantageous quality for methods development with AAV is its 60-fold icosahedral symmetry. That means that one can get to high-resolution with a relatively small data set. This quality allows one to assess different data collection conditions without having to collect large amounts of data.

#### 3.6.1 AAV and Tools for Assessing Data and Reconstruction Quality

AAV-DJ and the model sample GroEL were used to develop new metrics for assessing data and reconstruction quality for single particle cryo-EM. It was shown that there is a linear relationship between spatial frequency (the inverse of resolution) and the logarithm of the number of particles contributing to a 3D reconstruction, a so-called “ResLog” plot. Using AAV data, it was shown that the slope of a regression line fit to ResLog data corresponds to the quality of cryo-EM data, whereas the ResLog intercept corresponds to the quality of the reconstructed 3D map. It was shown that ResLog plots could thus evaluate the quality of cryo-EM reconstructions and potentially identify ones that result from artifactual single particle refinements. This analysis was taken a step further, and the AAV data was split into subsets according to data collection metadata to identify conditions that were significantly better or worse than the ResLog average. This revealed that ResLog analysis can be used to optimize conditions that promote the highest-quality reconstructions for a given sample.

#### 3.6.2 Reconstruction Refinement

AAV possesses several qualities that make it an attractive option for optimization of reconstruction refinement methods. First are its qualities that are of benefit to any single-particle project: high symmetry and large particle size. Large size and characteristic appearance facilitate particle picking, even at low defocus levels needed at high resolution, but AAV is not large enough to be particularly challenging during data processing. Of particular note in the case of the AAV-DJ data set,^128^ the diameter of AAV is large enough that the correction of microscope magnification anisotropy was critical to getting below 2.1 Å. This drove the project toward incorporating the magnification anisotropy correction of the Grigorrief group in early stages^311^ and later testing similar functionality as it was integrated into the newly released (at the time) Relion 3.1 refinement. The size of this particle was also large enough that Ewald sphere correction in postprocessing became critical to achieving the final ∼1.6 Å result, which arguably could be pushed somewhat further if this correction were integrated into the iterative refinement. The high-symmetry (and stable adherence to that symmetry) not only improves the throughput of single-particle averaging, but it makes orientation assignment more stable and accurate while simultaneously reducing the computational cost of processing (from a reduced search space).

A second useful property of AAV is from the extensive optimization of sample preparation. Because of this work, and the inherent properties of AAV, it is possible to produce samples that are stable and show extremely low amounts of background contamination when vitrified for cryo-EM. Furthermore, AAV can be sufficiently concentrated so that the particles in ice form pseudocrystalline 2D lattices in minimally thin ice. In particular, this maximizes both the quality of individual images and the throughput in number of particles imaged over time. Such dense particle packing has also been noted to help stabilize the sample better during imaging, which reduces signal lost to intraframe motion and also helps improve the amount of local signal for patch- or particle-based motion correction. Finally, and very important for the highest-resolution cryo-EM results, the high particle density greatly improves the signal available per image, and per wall-clock time, for estimating and correcting higher-order CTF aberrations.

#### 3.6.3 Atomic Refinement

With growth in biomolecular cryo-EM, one of the needs has been for refinement methods through which atomic models are improved by computationally optimizing the fit to the data while also best satisfying restraints and constraints to ensure that the structure adheres to *a priori* understanding of the principles of stereochemistry. Multiple approaches have been developed either from scratch or through adaptation of crystallographic methods, now directly, or indirectly improving the fit to the (real space) cryo-EM Coulombic potential map rather than the (reciprocal space) crystallographic (Fourier coefficient) structure amplitudes.^261,312−318^ These embody many algorithmic choices: (a) fitting metric: maximizing the map values at atoms, map correlations or least-squares residuals, (b) model parametrizations: individual-atom, torsion angle, or rigid group, (c) optimization algorithm: gradient descent or molecular dynamics, (d) types of stereochemical restraints and the weights with which they are applied, and so on.^319^ The choices impact the accuracy of structures and depend upon resolution, in terms of what can be discerned and the quality/quantity of data needed to define model parameters whose number depends on the parametrization. Yardsticks are needed to make these choices objectively. One strategy is to curate representative data sets released to developers for comparison of refinements, with sometimes a one-time opportunity for a blind challenge in fitting prepublication data with subsequent comparison to a published model.^320^ Another strategy uses data sets where ground truth structures are available. A blind challenge is unbiased, but there are no cross-validation methods for cryo-EM, so, without a known answer to measure coordinate accuracy, assessment is based on secondary metrics and can be confounded by overfitting. AAV is now a prime ground-truth test system.

Ground truth yardsticks are essential because measures of EM model accuracy remain under development and rudimentary. Within the field, there is heavy reliance on stereochemical assessments, which are necessary, but utterly insufficient because they are found to be orthogonal to measures of fit.^321^ Even at high resolution, it is usual practice in EM to apply all available stereochemical restraints, including (questionably) backbone torsion angles, thereby eliminating Ramachandran plots as a useful validation or quality metric.^321^

The requirement of a ground truth test is the availability of a structure determined at significantly higher resolution and/or by an experimental technique other than cryo-EM. Cryo-EM of AAV-2 started at nanometer resolution, which can be compared to the X-ray structure at 3 Å resolution.^1^ Structures at intermediate resolutions (∼5 Å) were pursued for AAV-DJ, for which there are not crystal structures. However, AAV-2 is highly homologous (62% sequence identity) and can be used to calculate an upper-limit estimate of error.^261^ It will be a good estimate if the real differences in structures are modest (on average) compared to the experimental errors of the test structure expected at the resolution. Refinements of ligand complexes can similarly be evaluated with the option of excluding, from assessment, small regions of the protein expected, *a priori*, to be different. At this point, the crystallographic yardsticks have been overtaken by higher resolution cryo-EM. Considering that they are only 62% identical in sequence, have been determined independently, and refined using different programs, the agreement between the 1.86 Å structure of AAV-2L336C and the 1.56 Å AAV-DJ is phenomenal: RMSD = 0.286 Å for all non-hydrogen atoms and 0.232 Å for C_α_ atoms. If equal error for both structures is assumed, these correspond to estimated errors below 0.2 Å and 0.16 Å (C_α_) within each structure. Expecting that well restrained refinements should be accurate to about one-fifth of the nominal resolution,^128,261^ these structures can be considered ground truths for refinements from low resolution to 2 Å. One caution is that, if now the yardstick models are also used as the starting point for test refinements, refinement protocols can appear perfect if the atomic model is not given the freedom to change.

At this point, the history of AAV cryo-EM is the advantage. A large number of structures are available (Tables 1–4) over a wide range of resolutions. Atomic models, built contemporaneously to the best of the experimenters’ abilities, can be used as realistic starting models for test refinements, unbiased by the now-known answer. This is a surprisingly unique resource. Because of more challenging alignment, cryo-EM structures of apoferritin are all beyond 4.7 Å resolution, and those worse than 2.5 Å were solved after determination of higher resolution crystal structures. GroEL represents an AAV-like wide array of cryo-EM resolutions, but high resolution EM structures were subsequent to the 2003 crystallography yardstick at 2 Å resolution.

This review is a testament to the recent gains in resolution of cryo-EM. It should be remembered that the median and average resolutions of EMDB submissions in 2020 were 3.5 and 6.3 Å, respectively.^322^ The majority of EM structures are at resolutions lower than those for which crystallographic refinement packages were designed or generally used. For these resolution regimes, best practices in refinement have not yet been established objectively. Rigorous testing with systems like AAV offers enlightening insights that will be key to maximizing the impact of cryo-EM in wide areas of structural biology.^261,319^

#### 3.6.4 Other

AAV is becoming more broadly appreciated as a well-characterized sample for electron microscopy, and so it is increasingly used as a test system in developing new EM approaches or applications. One recent example is in the application of liquid-phase EM of samples that are not flash frozen but diffusing in free solution.^323^ The approach might allow time-dependent studies using low electron doses and fast and sensitive cameras. While not (yet) rivaling the detail that can be seen with high resolution cryo-EM, few would previously have expected that much would be resolvable with ambient temperature beam damage, so this is an encouraging glimpse of what might be coming.

### 3.7 Process Optimization and Quality Control

Cryo-EM has applications throughout the lifecycle of an AAV-based therapeutic. Gene therapy projects in the preclinical stage benefit from the ability of cryo-EM to detect process-related impurities, such as residual nucleic acid, host cell proteins or debris, or traces of helper virus, if used. A number of analytical approaches to quantitate these impurities have already been established and qualified/validated, but each demands its own sample from an often precious and limited pool. As development of artificial intelligence-based methods for identifying particles observed in micrographs continues, there is the possibility that cryo-EM becomes an orthogonal analytical method for quantification of these impurities.^324^

Cryo-EM has made the most impact in quantification of empty, full, and intermediately packaged virions within a sample. Preparations of AAV are composed of virions that contain the entire intended genome (full particles); virions with no DNA packaged (empty particles), and virions encapsidating DNA of shorter length than the intended genome, either arising from fragments of the intended genome, host cell genome, or transfection plasmids (intermediate particles). The relative distribution of virions in a sample is a critical quality attribute, with therapeutic effectiveness directly related to the number of full particles.

Sedimentation velocity analytical ultracentrifugation (SV-AUC) is the current “gold standard” for quantification of relative packaging rates, with charge detection mass spectrometry (CDMS) being another option.^325,326^ Cryo-EM has the advantage of being much more readily available than CDMS and requiring much less material than SV-AUC. Cryo-EM also avoids staining artifacts that can often lead to inaccurate quantification associated with negative stain EM.

Cryo-EM has been shown to reach levels of accuracy similar to those of SV-AUC with ∼20 000 particles (150–200 micrographs), making cryo-EM also amenable to high throughput quantification on microscopes equipped with autoloaders. In their approach, Subramanian *et al.* used 2D and 3D classification methods to arrive at empty/full/intermediate populations and consumed less than 5% the amount of material that a typical SV-AUC run would require. Inherent in these classification-based approaches is a computational separation of intact AAV particles from product- and process-related impurities, which is a luxury that one is often not afforded by other methods.^134^

Cryo-EM’s advantages make it very suitable in quantification of empty/full/intermediate fractions during project development stages when material is in limited supply. It is also a great choice further along the pipeline, when high throughput is desired, during, for example, comparability exercises as part of FDA Investigational New Drug (IND) activity reports, Biological License Applications (BLA), or postcommercial filings.

Cryo-EM was also incorporated into a comprehensive comparative study, investigating the possibility that capsid post-translational modification might explain differences in transduction efficiencies of vectors produced in human and insect cells.^136^ For structure, AAV8 was used as the model system (Table 1), but no differences were detectable at 3.3 Å resolution between full and empty vectors and those produced by prevailing human (HEK293) and baculovirus/insect cell expression systems.^136^ Reliance on cryo-EM averaging within and between particles limits such approaches to modifications uniformly affecting all subunits. Thus, it is likely that analytical characterizations of post-translational modification will remain mostly in the domain of mass spectrometry.

## 4 Concluding Remarks and Outlook

It has been an exciting five years both for gene therapy and electron microscopy and doubly exciting to be at the confluence. The tangible impact of an FDA-approved effective treatment for spinal muscular atrophy (SMA^62−64^), a deadly progressive condition, has been realized through translational research built on many fundamental science foundations. One foundation has been the development of delivery vectors, and the structural virology that has illuminated AAV structure and host interactions. Advances in electron microscopy have seen it replace crystallography as the favored structural approach for viral capsids. It is now significantly easier, faster, and accessible to a wider cohort of investigators and usually providing results of equal or greater accuracy. Furthermore, complexes, that are not readily amenable to crystallization, may be terrific candidates for cryo-EM. Cryo-EM played a substantial role in understanding that AAV had multisite promiscuous and low specificity interactions with glycans and that the glycans should be reclassified as attachment factors rather than entry receptors.^233^ Cryo-EM then allowed interactions to be visualized with the newly found receptor, AAVR, and how the AAV5-like group differed from AAV4 and all other clades with a binding site for the AAVR PKD1 domain that is distinct from the PKD2-binding site of most serotypes. Comparisons with previous cryo-EM structures of antibody complexes at lower resolution have indicated potential competition between receptor and almost all neutralizing monoclonal antibodies whose complexes have been visualized, forcing a re-examination of the belief that mechanisms of neutralization were predominantly either inhibiting glycan interactions or were acting postcell-entry. Cryo-EM has been pivotal in recent reappraisals of the viral–host molecular interactions that underlie successful gene delivery.

The job is only part done. A number of partner host molecules have been identified, but their roles and interactions remain to be characterized both functionally and structurally.^119−121,233^ It is a sign of technical progress that the cryo-EM is no longer the bottleneck, but the prerequisite expression of usually poorly characterized cellular proteins in a form suitable for characterization of interactions with AAV. Recent structures have generated follow-on questions such as the meaning of distinct AAVR interactions between AAV5-like and other AAVs and the role of a highly conserved receptor in host cell specificity. Structure will be key in addressing these questions and others that only become apparent later.

Some questions would be better addressed if the structure of AAV could be visualized during the process of infecting a cell. New technology is coming online that enables just that. Focused ion beam-scanning electron microscopes are devices that enable milling of samples using a beam of charged ions, typically gallium ions. By directing a narrow beam of Ga^+^ at the sample, the sample in the path of the beam can be etched away. The Ga^+^ beam can be used to mill the sample into a thin lamella that is 300 nm or less, which is an appropriate thickness for imaging with a TEM. Recently it has been demonstrated that FIB milling can be performed on vitrified samples without damaging them, enabling researchers to mill cryogenically frozen cells.^327^ The cryogenically prepared lamella can then be imaged using tomography to yield high-resolution 3D reconstructions of samples of interest in their native state. This technology has recently been used to visualize assembly intermediates in mammalian orthoreovirus as it was maturing in infected cells.^328^ It is expected that this technology can be used to image AAV at different stages of infection, potentially revealing the molecular mechanisms AAV uses during infection. Of particular interest would be to capture images of AAV undergoing the presumed conformational changes taking place as it proceeds through and escapes endosomes.

AAV is subject to such comprehensive study because it is a foundation for the multitude of efforts toward the improved specificity, efficiency, and safety of *in vivo* gene delivery. Secondary benefits will be further prominence of AAV as a particularly thoroughly characterized model for fundamental virological questions. Many of the same types of virus–host interactions underly AAV’s role in gene delivery as would be important in understanding a pathogenic virus. One difference will be our motivation to modulate these interactions in engineering improved gene delivery. This will expose quickly the limits of our molecular understanding, and there will be an exciting phase of research where iterative cycles, involving both phenotypic and structural characterization, will forge a more robust understanding of viral–host interactions. Key to this will be the cryo-EM workflows emerging that can keep pace with laboratory discovery cycles.

## Abbreviations

AAV: adeno-associated virus
AAVR: AAV receptor (the gene product of KIAA0319L)
BEV: baculovirus expression vector
CC: correlation coefficient
CDR: complementary determining regions
CRISPR: clustered regularly interspaced short palindromic repeats
DDD: direct electron detection device
EM: electron microscopy
ET: electron tomography
Fab: antigen-binding fragment (of an antibody)
HEK: human embryonic kidney (cells)
HBD: heparin-binding domain
HSPG: heparan sulfate (HS) proteoglycan
IVIg: intravenous immunoglobulin
mAb: monoclonal antibody
MBP: maltose-binding protein
NAb: neutralizing antibody
PKD domain: polycystic kidney disease protein-like domain
PLA: phospholipase A
rAAV: recombinant AAV (vector)
rh: *Rhesus* macaque
rmsd: root-mean-square deviation
SIA: sialic acid
SOS: sucrose octasulfate
SPR: surface plasmon resonance
VP: viral protein
VP1u: N-terminal part of VP1 that is not shared with VP3
VR: variable region
wt: wild-type
XL-MS or x-MS: cross-linking mass spectrometry
